# EpiRegress: A Method to Estimate and Predict the Time-Varying Effective Reproduction Number

**DOI:** 10.3390/v14071576

**Published:** 2022-07-20

**Authors:** Shihui Jin, Borame Lee Dickens, Jue Tao Lim, Alex R. Cook

**Affiliations:** 1 Saw Swee Hock School of Public Health, National University of Singapore and National University Health System, # 10-01 Tahir Foundation Building, 12 Science Drive 2, Singapore 117549, Singapore; shihui.j@nus.edu.sg (S.J.); ephdbsl@nus.edu.sg (B.L.D.); juetao@nus.edu.sg (J.T.L.); 2 Department of Statistics and Data Science, National University of Singapore, Singapore 117546, Singapore

**Keywords:** Bayesian inference, COVID-19, epidemic control, regression, reproduction number

## Abstract

The time-varying reproduction ($R_{t}$) provides a real-time estimate of pathogen transmissibility and may be influenced by exogenous factors such as mobility and mitigation measures which are not directly related to epidemiology parameters and observations. Meanwhile, evaluating the impacts of these factors is vital for policy makers to propose and adjust containment strategies. Here, we developed a Bayesian regression framework, EpiRegress, to provide $R_{t}$ estimates and assess impacts of diverse factors on virus transmission, utilising daily case counts, mobility, and policy data. To demonstrate the method’s utility, we used simulations as well as data in four regions from the Western Pacific with periods of low COVID-19 incidence, namely: New South Wales, Australia; New Zealand; Singapore; and Taiwan, China. We found that imported cases had a limited contribution on the overall epidemic dynamics but may degrade the quality of the $R_{t}$ estimate if not explicitly accounted for. We additionally demonstrated EpiRegress’s capability in nowcasting disease transmissibility before contemporaneous cases diagnosis. The approach was proved flexible enough to respond to periods of atypical local transmission during epidemic lulls and to periods of mass community transmission. Furthermore, in epidemics where travel restrictions are present, it is able to distinguish the influence of imported cases.

## 1. Introduction

The combination of non-pharmaceutical interventions (NPIs) such as border controls, social distancing, and test-trace-isolate-quarantine systems allowed a number of countries and regions in the Western Pacific to suppress COVID-19 transmission for extended periods [1]. During periods of low but non-zero community transmission, it can be difficult for policy makers to make sense of disease transmission potential. Consequentially, it remains difficult to ascertain the excessiveness of contemporaneous control measures, which are economically and socially costly, and whether outbreaks represent nascent waves or stochastic flare-ups of disease transmission. These uncertainties are further magnified when secondary community infectees result from imported, active infectors [2].

A commonly used metric of disease transmissibility is the instantaneous or effective reproduction number $R_{t}$, which is defined as the ratio of the number of new local infections generated at time t and the total infectiousness of infected individuals at that time [3]. As an indicator of disease transmissibility, the threshold of unity signals if the epidemic is growing. Given its utility to policy makers for epidemic assessment, $R_{t}$ has been used to understand the impact of public health interventions for outbreaks caused by pathogens such as smallpox, influenza, severe acute respiratory syndrome coronaviruses 1 and 2 [3,4]. Over the past decade, several approaches have been proposed to estimate $R_{t}$ that extend the seminal Wallinga and Teunis method [5], including EpiEstim by Cori et al., EpiFilter by Parag and EpiInvert by Alvarez et al. [3,6,7]. Although these methods pool information across time to improve the precision and hence the utility of $R_{t}$ estimates, they do not account for exogenous factors that may substantially affect transmissibility—such as mobility data or characteristics of the NPIs that took place over time. Exogenous factors may provide additional information on disease transmissibility, especially when the number of cases is low and uninformative.

Furthermore, despite improvements in EpiEstim and EpiFilter being proposed to distinguish imported cases from local ones [8,9], when quarantine measures on international travelers are in place, the risk of secondary transmission from imported cases may differ substantially from that of the community, and when imported cases constitute a substantial fraction of the total number being detected, it is important for estimates of $R_{t}$ to account for heterogeneous transmissibility between groups.

Therefore, this study outlines a method to combine case time-series, stratified by importation status, and covariates (vaccination rates, mobility levels, and policy implementation) to estimate $R_{t}$ in a regression-style framework. We applied the method to COVID-19 in four Western Pacific countries and regions that had both extended periods of low and high community incidence. The method we developed, EpiRegress, is completely data-driven, assigning little prior information to $R_{t}$s. It allows changes in historical $R_{t}$ to be explained through relevant, exogenous covariates, and thereby provides reliable nowcasts of the current $R_{t}$ despite the possible absence of case counts contemporaneously. The approach therefore can provide policy makers real-time estimates of disease transmission potential to guide decisions on containment measures.

## 2. Methods

In brief, the EpiRegress framework assumes a negative binomial relationship between daily case counts and $R_{t}$, where the number of cases on a certain day had an expected value equal to the sum of local and imported infectiousness. We estimated $R_{t}$ over time by fitting case counts in a model akin to a generalised linear regression where dependent variables were taken to be mobility, epidemiological, and policy data. The Metropolis–Hastings algorithm was then used to derive the joint posterior distribution of the parameters before predictions on case count were made based on parameter estimates. We exemplified the utility of our approach through application to four Western Pacific countries or regions (henceforth denoted regions)—New South Wales, Australia; New Zealand; Taiwan, China; and the city state of Singapore—over the period from January to September 2021. These four regions and this epoch were selected as they had periods of low incidence, making estimation of $R_{t}$ harder due to uninformative case data. The datasets utilised and methods developed are discussed below.

### 2.1. COVID-19 Data 

Reported COVID-19 case counts in New South Wales, New Zealand, Singapore, and Taiwan were collected from the government health websites of these four regions respectively [10,11,12,13]. They were extracted from 12 November 2020 (50 days prior to year 2021) to 30 September 2021, for the four regions. Local cases and imported cases were separated for the regions New South Wales, New Zealand, and Taiwan while for Singapore, we excluded cases reported in foreign worker dormitories for both local and imported cases as they had a distinct disease epidemiology due to localized movement restrictions and denser living conditions compared to the community populace (Figure S2) [14,15]. In the Supplementary Information, we demonstrate that these exclusions do not significantly affect $R_{t}$ estimates (Figure S9).

The serial interval distribution was derived based on the time between notification events of 157 pairs of primary and secondary household cases in Singapore in 2021 [16]. We approximated the empirical serial interval distribution with a discrete, truncated log normal distribution using a mean of 4.1 days and standard deviation of 3.5 days (Figure S3). We used these data to estimate the infection potential assuming that the majority of the local cases were constituted of the B.1.617.2 variant (henceforth denoted Delta variant) [17], and similar nonpharmaceutical interventions had the intended effects of facilitating early detection, isolation of new cases, and limiting spread [18].

We also included the time-varying proportion of cases of the Delta variant as a candidate factor to explain changes in $R_{t}$ using data from GISAID; for New South Wales, the Australian proportion was used as no state-level proportion was available [19]. This inclusion was to account for the variant having a significantly higher basic reproductive number than the original virus [20]. For all the regions but Taiwan, the proportion rose gradually from 0 in mid-March to nearly 100%, while for Taiwan it rapidly grew from 0 to 100% in mid-June. The proportion was 100% for all the four regions from mid-July to the end of September 2021 (Figure S1). The time window predated the emergence of the Omicron variant of SARS-CoV-2.

### 2.2. Policy Data

Policies introduced in the four regions were extracted from the Oxford COVID-19 Government Response Tracker (OxCGRT) [21], which has provided information of government responses to COVID-19 around the globe since 1 January 2020. The dataset consists of 16 different indicators grouped into categories of closures and containment (c), economic response (e), and health systems (h), together with four indices calculated as functions of individual indicators from 1 January 2020. Detailed descriptions of these indicators and indices were listed in the Supplementary Information (Table S1). We extracted data until 30 September 2021 and imputed each of the missing values (0.3%) with the last available value in that column. For Singapore, we introduced six more variables representing discrete intervention phases in place from November 2020, to September 2021, including Phase 2, Phase 3, Phase 2 (Heightened Alert), Phase 3 (Heightened Alert), Preparatory Stage, and Stabilization Phase (taken as a reference variable) [22], during which the government adopted distinctive containment strategies on workplaces, social gatherings, dining, and entertainment facilities.

Variations in stringency, government response and containment health index values were almost the same for each specific region while for economic support index, only New South Wales and Taiwan recorded a sudden change by the end of March and May respectively. The greatest change in the first three index values took place in mid-August for New Zealand, and in mid-May for Taiwan. Changes in these indices tended to be milder for New South Wales and Singapore. Variations in the policy indicators, by comparison, were not so large and many remained constant throughout the nine-month period. Generally, indicators belonging to the same category had similar changes and the trends were mostly reflected in the corresponding indices (Figure S1). 

### 2.3. Google Mobility Data

Mobility data (Figure S1) for the four regions were obtained from Google’s Community Mobility Reports from 12 November 2020 to 30 September 2021 [23]. The data reflect relative changes in time from a pre-COVID-19 outbreak baseline that visitors spend in six different types of places: residential, workplace, retail and recreation, grocery and pharmacy, parks, and transit stations. Generally, the time people spent in the last five types of places were strongly and positively correlated with each other and negatively correlated with the time people spent in residential areas, though the trend between parks and other types of places for New South Wales and New Zealand were not so obvious (even converse). Variation in time spent in workplaces was the largest for all the four regions and a prominent weekly circle was observed while substantial changes in the variables were seen in May to August (Figure S1).

### 2.4. Vaccination Data

The vaccination doses administered per 100 people in New Zealand, Singapore, and Taiwan were collected from covidvax.live [24], an online platform that provides real-time statistics on vaccine doses registered worldwide. We obtained daily doses administered for New South Wales from COVID LIVE [25], an Australian website whose data sources are media releases and state health departments, and took the population of the region to be 8.2 million. The time range for vaccination data was from 1 January to 30 September 2021. For simplicity, we calculated the ‘vaccination rate’ as half of the average vaccination doses administered per person, i.e., $0.5\;D_{ti}$, where $D_{ti}$ is the mean vaccination doses administered per person by time t in region i. Vaccination started the earliest in Singapore, rose at almost a constant speed from March to July and slowed down from August as the vaccinated reached around 80% of the population. For both New South Wales and New Zealand, the rate started to rise from late March and accelerated significantly from mid-July. Taiwan, however, is the region with lowest vaccination rate among the four, where few people were vaccinated by mid-June and, by the end of September, only around 40% of the population had been vaccinated (Figure S1).

### 2.5. Modelling Daily Number of Cases and Time-Varying Reproduction Number

Using the serial interval probability mass at s days, $w_{s}$, and the number of reported cases before day t, $I_{1:t-1}$, the number of local cases on day t, $I_{t}^{local}$, is assumed to follow a negative binomial distribution with mean

(1) $$\mu_{t}=\overset{t-1}{\underset{u=t-\Delta t}{\sum }}I_{u}^{local}w_{t-u}R_{u}+\phi \overset{t-1}{\underset{u=t-\Delta t}{\sum }}I_{u}^{imported}w_{t-u}$$

and variance

(2) $$\sigma_{t}^{2}=\tau \mu_{t},$$

where ϕ is assumed to be the constant risk of transmission per imported case into the community, τ is the inflation factor for the variance, Δt is the length of the time window $\left[t-\Delta t,\;t-1\right]$ when a primary case is likely to cause a secondary case and for each day u, $I_{u}=I_{u}^{local}+I_{u}^{imported}$ and $R_{u}$ is the instantaneous reproduction number aforementioned. Allowing for enough time between neighboring generations of infections [26], we truncated the serial interval at Δt=50 for computational purposes by setting

$$\overset{50}{\underset{t=1}{\sum }}w_{t}=1$$

and discretized the serial interval distribution by letting

$$w_{t}=f\left(t\right)/\overset{50}{\underset{s=1}{\sum }}f\left(s\right),$$

where $f\left(\cdot \right)$ is the probability density function of the log normal distribution with mean 4.1 days and standard deviation 3.5, which is the aforementioned approximation of the empirical serial interval distribution (Figure S3).

### 2.6. Augmenting $R_{t}$ Inference with Exogenous Factors

We assume that $R_{t}$ can be explained by a series of exogenous factors at time t, thus:

log R=Xβ+α,

where $X_{t\times p}$ is a matrix with p exogenous factors measured across time points 1,2,3,⋯, t, $\beta ={\left(\beta_{1},\beta_{2},\cdots ,\beta_{p}\right)}^{T}$ a vector of time-invariant coefficients, α a constant intercept, and $R={\left(R_{1},R_{2},\cdots ,R_{t}\right)}^{T}$ a vector of time-varying reproduction numbers. Covariates with constant values were excluded.

Since there are a large number of covariates β that may potentially affect or be correlated with $R_{t}$, we make use of the Bayesian Lasso [27] for parameter selection by assigning a Laplace prior distribution with mean 0 and variance $2\lambda^{-2}$ for each entry of β, i.e.,

$$\beta_{i}∼Laplace\left(\lambda^{-1}\right),i=1,2,\cdots ,p,$$

where λ≥0 is the penalty in the $L_{1}$-penalized least square error function

$${\left(\tilde{R}-X\beta -\alpha \right)}^{T}\left(\tilde{R}-X\beta -\alpha \right)+\lambda \overset{p}{\underset{i=1}{\sum }}\left|\beta_{i}\right|,\;\tilde{R}=\log\;R-\bar{\log\;R}1_{n},$$

which the Lasso estimates minimize.

We set λ=5 (see Table S3 for more details). We then used an auto-regressive Metropolis–Hastings algorithm to estimate the joint posterior distribution of the parameters $\Theta =\left(\alpha ,\beta ,\phi ,\tau \right)$ and thus those of $R_{t}$s with a Gaussian proposal distribution

$$\Theta^{new}∼N_{p}\left(\Theta^{old},V_{\Theta }\right),$$

and each new draw of parameters $\Theta^{new}$ was accepted with probability

$$\min\left(1,\frac{\prod_{t}P\left(I_{t}|I_{1:t-1},\Theta^{new},\lambda ,X\right)P\left(\Theta^{new}\right)}{\prod_{t}P\left(I_{t}|I_{1:t-1},\Theta^{old},\lambda ,X\right)P\left(\Theta^{old}\right)}\right),$$

where $P\left(I_{t}|I_{1:t-1},\Theta ,\lambda ,X\right)=f_{\mu_{t},\tau }\left(I_{t}\right)$ is the conditional likelihood and $P\left(\Theta \right)=h\left(\alpha \right)1_{\left\{\tau >0\right\}}1_{\left\{\phi >0\right\}}\prod_{i}g_{\lambda }\left(\beta_{i}\right)$ the prior distribution of the parameters. In these, $f_{\mu_{t},\;\tau }\left(\cdot \right)$ is the probability mass function for negative binomial distribution with mean $\mu_{t}$ and variance $\tau \mu_{t}$, $g_{\lambda }\left(\cdot \right)$ is the density function for Laplace distribution with location parameter 0 and scale parameter λ, $h\left(\cdot \right)$ is the density function for the non-informative normal prior $N\left(0,100^{2}\right)$ which we assigned to the intercept α, while $1_{\left\{\tau >0\right\}}$ and $1_{\left\{\phi >0\right\}}$ are the positive constraints (indicator function taking 1 if and only if the argument of the function is positive) we set for τ and ϕ respectively. 

We standardized the X matrix before doing regression and excluded covariates which remained constant throughout the inference window to allow for the comparison of different entries of β.

To examine the roles of different factors in accounting for changes in $R_{t}$s, three model variants with different factors included in the covariate matrix X were considered: (i) a full model that included all available factors (Table S2), (ii) a model excluding policies that included only mobility and epidemiological factors, (iii) a hybrid model that included ‘retail and recreation’ and ‘residential’ from google mobility data, vaccination rate and all indicator covariates in the Oxford policy data except ‘testing policy’ and ‘vaccination policy’. The second model variant was chosen to see if mobility and epidemiology variables could fully reflect changes in policy-related variables, making the latter redundant in $R_{t}$ estimation. Variables in the last model variant were selected based on the correlation matrixes of Xs in the full model, i.e., some of the variables with correlation coefficients close to 1 were excluded in the hybrid model. 

To compare the fits of different model variants, we used the Deviance Information Criterion (DIC), which measures the deviance while penalizing model complexity. The formula for calculating the DIC is

$$DIC=\bar{D\left(\Theta \right)}-\frac{1}{2}\bar{Var\left(D\left(\Theta \right)\right)},$$

where $D\left(\Theta \right)=-2\log\left(\prod_{t}P\left(I_{t}|I_{1:t-1},\Theta ,\lambda ,X\right)\right)+C$, $\Theta =\left(\alpha ,\beta ,\phi ,\tau \right)$ is the collection of the parameters to be estimated and C is some constant. 

### 2.7. Simulation for Validation of the Method

Since the true $R_{t}$ values are not observable in the case studies, we used simulations to validate the proposed approach. We considered two different X covariate matrices over a window of 230 days (henceforth, Scenario 1 and Scenario 2): one taken directly from the mobility, epidemiological, and policy data of New Zealand between 1 January and 28 June 2021, and the other with 20 randomly generated covariates, among which 6 are continuous variables and the rest 14 are ordinal (range for each variable: 0–4). Similar to inference performed in case studies, we standardized the X matrices and excluded the covariates with constant values, after which the first X covariate matrix was left with 21 variables.

We randomly generated 4 different sets of β coefficients for each scenario, calculated the corresponding $R_{t}$s and further simulated local case counts for 230 days from the negative binomial distribution with mean and variance specified in Equations (1) and (2) in Section 2.5 previously. Imported case counts were obtained from a discrete uniform distribution with left end as 0 and right end as a number in the set $\left\{10,\;15,\;20,\;30\right\}$. The constant risk of transmission per imported case into the community ϕ was set to be a fixed value of 0.01 and the inflation factor for the variance, τ, was taken in the range 3–6. Utilising the simulated incidence curves, we estimated $R_{t}$s with EpiRegress over a window of n=180 days (i.e., the likelihood function for estimating $R_{t}$s was $P\left(I_{51:230}|I_{1:229},\;X,\beta ,\alpha ,\;\phi ,\tau ,\lambda \right)$) and compared them with the ‘real’ values by calculating the mean absolute errors (MAE) and mean absolute percentage errors (MAPE) as follows:

$$MAE=\frac{1}{n}\sum_{t=1}^{n}\;\left|{\hat{R}}_{t}-R_{t}^{sim}\right|$$

and

$$MAPE=\frac{1}{n}\sum_{t=1}^{n}\frac{\;\left|{\hat{R}}_{t}-R_{t}^{sim}\right|}{R_{t}^{sim}},$$

where ${\hat{R}}_{t}$ is the posterior median and $R_{t}^{sim}$ is the simulated $R_{t}$ for day $\left(t+49\right)$ in the original dataset. We also calculated the successful coverage rates (SCR) for the proportion of the time in the 180 days where the simulated $R_{t}$ values, $R_{t}^{sim}$, fell within the 95% CrIs of the estimated $R_{t}$s.

Using the same incidence curves, we additionally performed $R_{t}$ estimations with EpiEstim, EpiFilter, and EpiInvert for comparison purposes, but we only calculated MAEs and MAPEs for point estimates by EpiInvert as the relationship between $R_{t}$ and $I_{t}$ which it uses in the renewal equation is the same as the that for simulating the case counts. 

### 2.8. Prediction of Case Counts

If values of the covariates on day t, $X_{t}=\left(X_{t1},X_{t2},\cdots ,X_{tp}\right)$, are available, EpiRegress enables us to estimate the number of local cases on day $\left(t+1\right)$, $I_{t+1}^{local}$. This is done in two steps. First, we obtain the posterior predictive distribution of $\log\;R_{t}=X_{t}\beta +\alpha$ by doing MCMC simulations with a shifting window of 90 days, i.e., using data over the time interval $\left[t-89-\Delta t,t\right]$ to calculate the likelihoods for the case counts from day $\left(t-89\right)$ to day t, where Δt=50 is the maximum length of serial interval aforementioned. Since $I_{t+1}^{local}$ follows a negative binomial distribution with mean and variance as in (1) and (2), we obtain the posterior predictive distribution of $I_{t+1}^{local}$ by performing Monte Carlo sampling. Note that past data are used to generate these samples, rather than past simulations, so the results presented represent nowcasting accuracy rather than long-term predictions, which would in any case require future covariates to be predicted. 

Analyses were conducted in R [28] and C++.

## 3. Results

### 3.1. Validation of $R_{t}$ Estimation through Simulation

We simulated four incidence curves from each of the two covariate matrices, estimated the $R_{t}$s with EpiRegress, EpiEstim, EpiFilter, and EpiInvert and compared the estimates with the simulated ‘true’ values (Figures S4 and S5, Table 1). All but EpiInvert successfully produced $R_{t}$ estimates for all scenarios; EpiInvert failed for the third simulated time series of case counts in Scenario 1 when the maximum number of local cases reported in a day was below 100. The same method also failed or gave negative $R_{t}$ estimates when the number of local cases remained low and imported cases were excluded in inference for $R_{t}$.

Generally speaking, compared with EpiEstim and EpiFilter, estimates by EpiRegress only had significantly larger credible intervals when there were zero or single-digit case counts for many consecutive days (e.g., Figures S4g,h and S5f,h), but the uncertainties allowed for rises and falls in the simulated ‘true’ $R_{t}$s to be better captured by EpiRegress, even in the absence of incidence data. This is demonstrated by the significantly larger SCRs for the estimates by EpiRegress, which were all around 95%, the percentage where true values are expected falls within the estimated intervals.

Additionally, the deviation of EpiRegress’s point estimates from the ‘true’ values, in terms of MAE and MAPE, were also the smallest among the diverse methods for all four simulations in both scenarios (Table 1). For EpiRegress, MAEs for Scenario 1 were generally no larger than 0.25 and for Scenario 2, they were all around 0.35. MAPEs were smaller than 0.30 for all but one simulation but that ‘outlier’ was caused by an extremely small simulated $R_{t}$ (<0.001) and if we excluded the corresponding time point by averaging the absolute percentage errors over the rest of the time points, the MAPE for that simulation decreased to 0.007.

### 3.2. Estimation of $R_{t}$ for Four Regions in the Western Pacific

**Figure 1:**
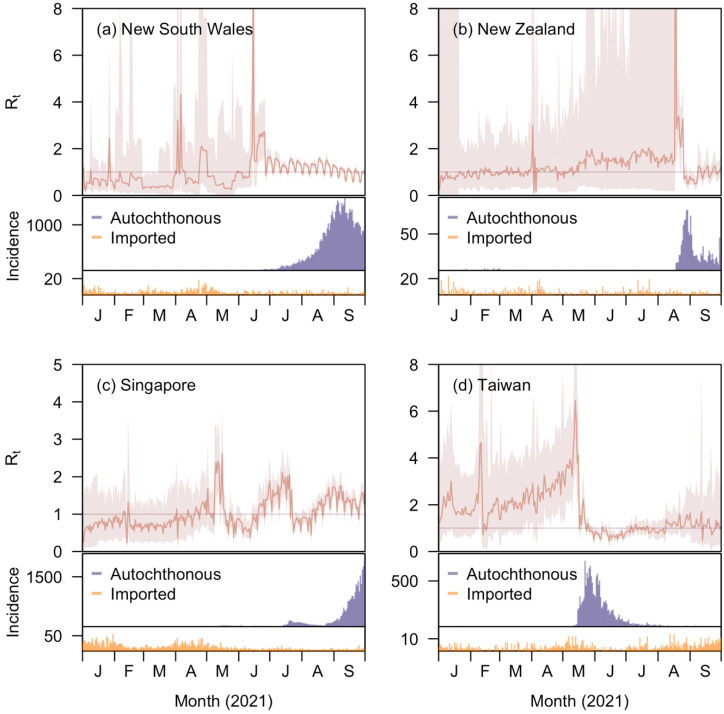
Estimates of time-varying effective reproduction number, $R_{t}$, across time (red), together with reported numbers of autochthonous (blue) and imported (orange) new cases, in (**a**) New South Wales; (**b**) New Zealand; (**c**) Singapore; (**d**) Taiwan by EpiRegress using all the covariates available (i.e., the full model). Reported cases residing in foreign worker dormitories were excluded for both local and imported cases in Singapore.

We estimated the time-varying effective reproduction number from 1 January to 30 September 2021 on a daily basis for New South Wales, New Zealand, Singapore, and Taiwan. On average, the time-varying effective reproduction number $R_{t}$ is centred around 1 with fluctuations at different times (Figure 1).

The estimates of $R_{t}$ were responsive to regional outbreaks despite the restrictions imposed by the regression structure. In New South Wales, the low and sub-critical transmissibility during the first six months of 2021 were punctuated by short periods of elevated $R_{t}$ corresponding to the occasional emergence of local cases. This period gave way starting from July 2021, when a large community wave of the Delta variant emerged with $R_{t}$ averaging around 1.16 (IQR: 0.98–1.37). New Zealand and Taiwan both also experienced a lengthy period of low community incidence during which $R_{t}$ hovered at 1 for New Zealand and above 1 for Taiwan. In Taiwan, the $R_{t}$ fell greatly by over 5 in four days after the epidemic wave emerged and peaked in mid- and late-May, and then fell further to lower levels than the pre-wave era with posterior medians concentrated around 1. The $R_{t}$ in Singapore largely remained below 1 at a median of 0.76 (Interquartile Range [IQR]: 0.66–0.88) before 24 April 2021. In the three subsequent epidemic waves, the posterior medians of $R_{t}$ in Singapore rose sharply to over 1 and in the large Delta wave that started in August, $R_{t}$ estimates remained around 1.3 (IQR: 1.08–1.45).

### 3.3. Importation Effect

**Figure 2:**
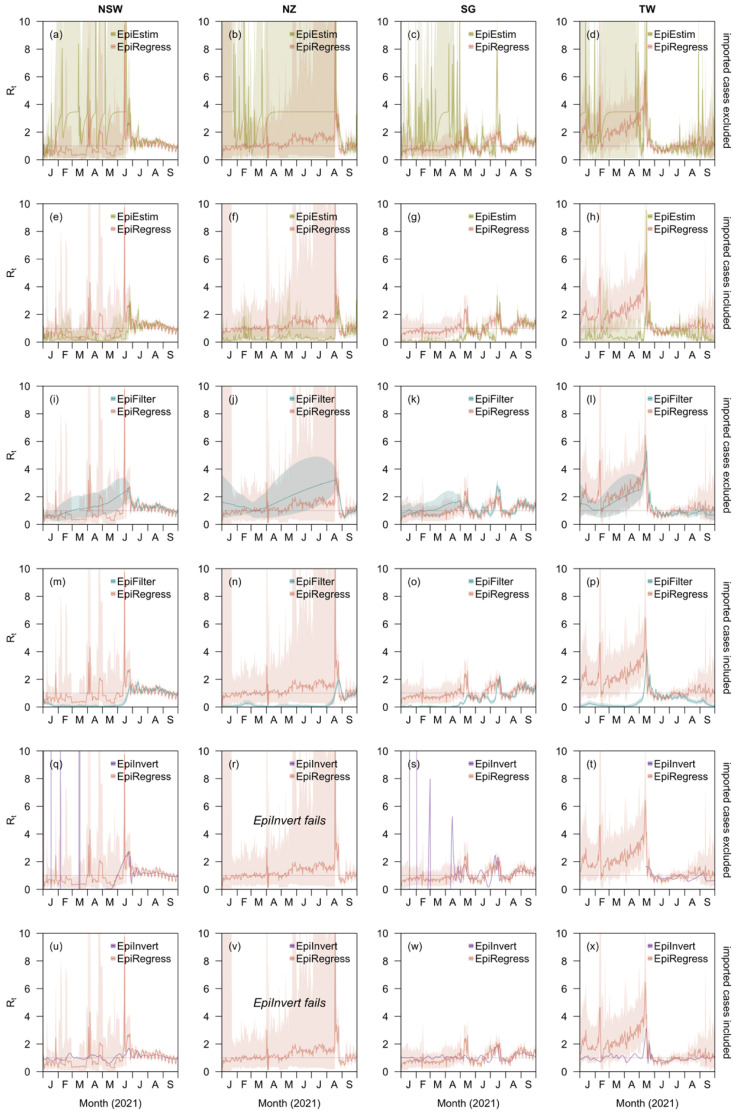
Comparison of EpiRegress against alternative $R_{t}$ estimation methods (**a**–**d**) EpiEstim with imported cases excluded, (**e**–**h**) EpiEstim with imported cases included, (**i**–**l**) EpiFilter with imported cases excluded, (**m**–**p**) EpiFilter with imported cases included, (**q**–**t**) EpiInvert with imported cases excluded and (**u**–**x**) EpiInvert with imported cases included for New South Wales (column 1), New Zealand (column 2), Singapore (column 3), and Taiwan (column 4). EpiInvert failed to perform $R_{t}$ estimation for New Zealand when imported cases were either included or excluded in the total case counts.

Imported cases in all four regions made limited direct contributions to the number of cases with the average imported case resulting in 0.04 (95%CrI 0.02–0.07) local cases in New South Wales, 0.02 (0.01–0.04) in New Zealand, 0.03 (0.01–0.06) in Singapore, and 0.10 (0.03–0.22) in Taiwan. Despite the small impact on transmission, accounting for imported cases was important to estimate the local effective reproduction number, as can be seen in the comparison of our method with EpiEstim, EpiFilter, and EpiInvert—none of which treats effects of imported and local cases on local transmission differently (Figure 2). To estimate $R_{t}$ using either EpiEstim or EpiFilter, we conducted two analyses wherein we either removed imported cases from the analysis entirely or assumed equal transmissibility of imported cases as local ones. The choice of whether to count imported cases towards the denominator for the effective reproduction number had a substantial impact on estimates when imported cases constituted a sizable proportion of the total caseload. In such time periods, treating the impact of imported cases the same as that of local cases in both EpiEstim and EpiFilter led to estimates of $R_{t}$ below the critical threshold of unity (Figure 2e–h,m–p), whereas excluding them from the analysis led to estimates above unity (Figure 2a–d,i–l). While the inclusion of the imported cases generates smaller credible intervals with less uncertainty for both EpiEstim and EpiFilter, the intervals obtained when excluding these cases are more likely to cover estimates by our method, which explicitly accounts for differences in effects on local case counts between the two case types, when those of local cases were over ten times more substantial than those of imported ones according to our estimation. In all of the comparisons, estimates were comparable for the three models when local case counts were over 30 and imported cases only accounted for fewer than 20% of total case counts. In addition, for EpiInvert, two similar analyses were also performed (Figure 2q–x) with the only difference being that when imported cases were included, they were simply counted as part of the daily cases, i.e., $I_{t}=I_{t}^{local}+I_{t}^{imported}$, as the approach does not distinguish these two case types or consider the fact that imported cases cannot be infected by past local cases. EpiInvert however failed to generate $R_{t}$ estimates for New Zealand where continuous zero local case counts were recorded for a continuous period of 148 days and for the remaining regions. It also failed to produce positive $R_{t}$ estimates for all the time points when imported cases were not included as part of the cases. We therefore did not compare estimates by EpiInvert with those by EpiRegress, though we still visualized the differences in Figure 2.

### 3.4. Validation of Coverage

Using the posterior distributions of the parameters, we derived the posterior predictive distribution of the mean and variance for each day’s local cases, based on which we estimated the 50% and 95% credible intervals for the case counts. The percentages of covering of the true values for both two types of intervals by which all exceeded the expected values, were 50% and 95%, respectively (Figure S6, Table S4)

**Figure 3:**
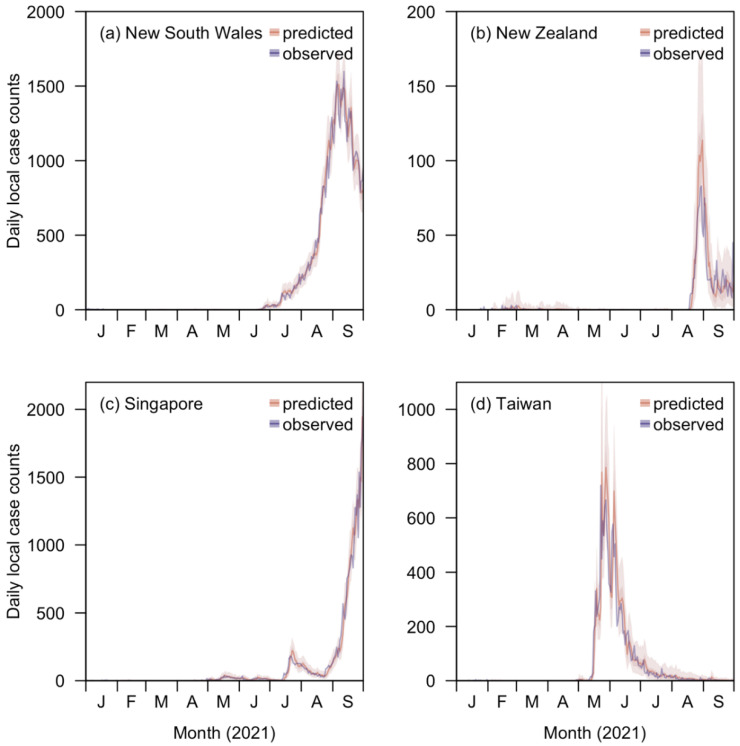
Comparison of the reported local case counts with the case counts forecasted (one day ahead) with the posterior predictive distribution of time-varying effective reproduction number, $R_{t}$, in: (**a**) New South Wales; (**b**) New Zealand; (**c**) Singapore; (**d**) Taiwan, estimated by EpiRegress using all the covariates available (i.e., the full model).

To evaluate the nowcasting function of EpiRegress, we further forecasted local case counts over a window of 240 days, from 3 February to 30 September 2021, when the observed local case count for at least one region was greater than 0 per week. A 90-day inference window was utilized to simulate the posterior draws of the parameters involved, which were then used to derive the posterior predictive distributions of the following 7 day local cases. While the proportion of the actual case counts falling within the 95% posterior predictive distribution interval might slightly decrease as the time between the forecasting day and the end of the inference window increased from one to seven days, all were close to the expected coverage rate of 95% (Figure 3, Table S5), indicating good adherence to advertised coverage. 

### 3.5. Robustness of EpiRegress to Covariate Choice

We compared $R_{t}$ estimates given by different selections of covariates with the three model variants: (i) using all covariates, (ii) excluding policy factors, and (iii) using ‘retail and recreation’ and ‘residential’ from google mobility data, vaccination rate, and all the indicators but ‘testing policy’ and ‘vaccination policy’ in the Oxford policy data (Table S2). To assess the fits of the models, we calculated DICs for each set of results and found smaller models usually had greater DICs, meaning a larger model with more covariates would produce better estimates of $R_{t}$ despite the additional complexity (Table S6). However, comparing posterior medians (Figure S7, Table S7) shows that $R_{t}$s were mostly determined by mobility and epidemiological covariates (Table S1) and were robust to the changes in the model variants in New South Wales, Singapore, and Taiwan within the period from July to September when local case counts were relatively high. The introduction of more factors in the full model, despite possible high correlations, appeared to cause EpiRegress’ $R_{t}$ point estimates to lower by around 1 and become more sensitive to variations in the local case counts when there were few local cases reported. For New Zealand, in particular, which had the longest duration of low numbers of local cases, the differences of $R_{t}$ estimates between the three versions were more substantial. Otherwise, the measure of smoothness (Table S8) suggested that the different choices of covariates had limited effect.

### 3.6. Impact of Distinct Covariates

**Figure 4:**
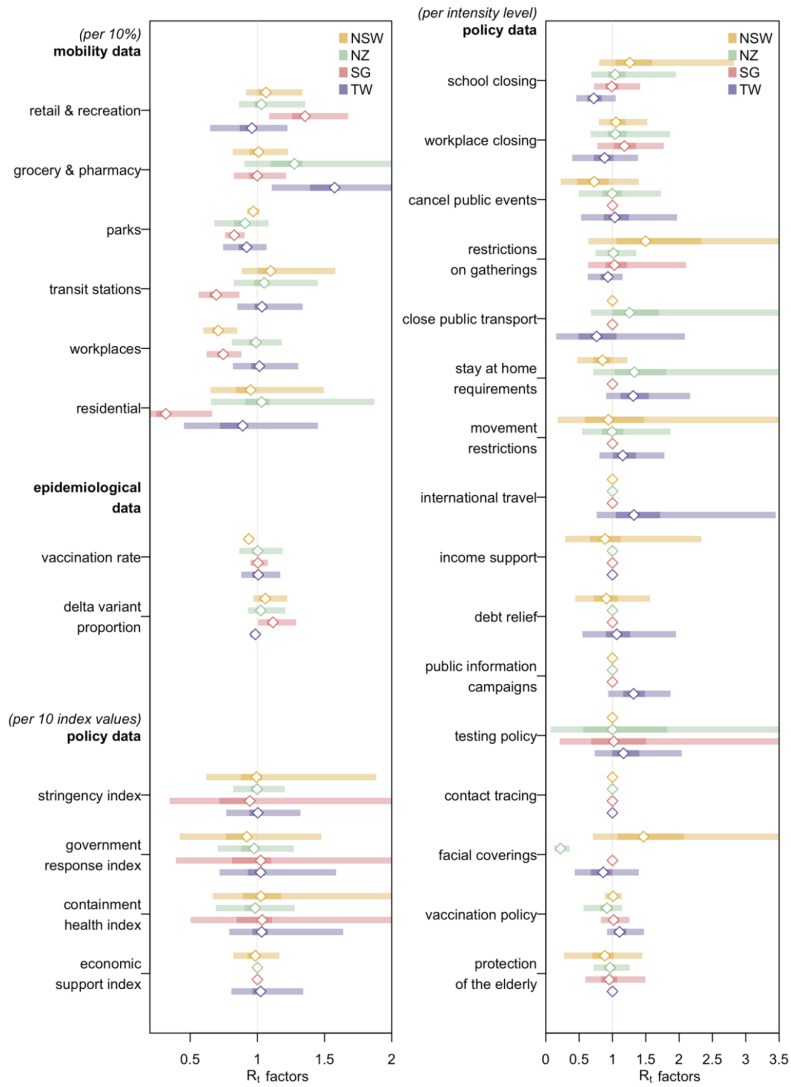
Impacts of different factors on $R_{t}$ (posterior medians, 50% and 95% credible intervals) for the four different regions in the full model with all the covariates available. Mobility factors were measured as percentage changes compared to a pre-COVID-19 outbreak baseline for each region; Vaccination rate refers to the proportion of the vaccinated population and policy indicators were ordinal variables indicating intensity levels, each ranging from zero (the least severe) to a maximum of five (the most severe). Factors related to different phases in Singapore were excluded from the model in this comparison of different factors’ effects on $R_{t}$ (Figure S8).

We assessed the effects of different factors on transmission potential based on the full model (Figure 4). From the point estimates of the contributions, an increase in the time people spent at retail and recreation or grocery and pharmacy was generally associated with a higher $R_{t}$ whilst a rise in time spent in parks or residential places was correlated with a drop in $R_{t}$. On average, a 0.7% decrease in $R_{t}$ came with every 1% increase in vaccination rate in New South Wales, which was not observed for the other three regions. The Delta variant proportion was closely related to an increase in $R_{t}$ with the most significant effect appearing in Singapore where a 1% increase in the proportion raised $R_{t}$ by 1.1% (0.1–2.5%). Though its influence in Taiwan appeared to be negative at −0.2% (−0.5% to +0.1%), this unexpected observation could be accounted for by the fact that the epidemic wave in Taiwan had almost come to an end with the arrival of the Delta variant cases in the area. For policy-related factors however, the effects on transmission potential varied from region to region, mostly centered around 1 with large 95% credible intervals due to the high aforementioned correlations. Nonetheless, there were still some noticeable effects. For instance, in New Zealand, each level’s increase in the intensity of measures regarding facial covering brought down $R_{t}$ by 78% (64–87%) whilst the per level’s increase in intensity of measures related to school closing in Taiwan saw a 28% (−5% to +54%) decrease in $R_{t}$. 

## 4. Discussion

Over the course of the COVID-19 pandemic, the time varying reproduction number, $R_{t}$, has received considerable public attention as a metric of the waxing and waning of epidemic trajectory. Examples include Wuhan, China [4] and diverse European countries [29]. When daily case counts are large, standard methods to estimate $R_{t}$ are successful, though they may face data challenges with complications such as day of the week effects [30]. This problem is mitigated by EpiInvert with its signal processing approach [7]. However, in places and times where disease transmission is low, having small numbers of cases either makes the methods fail (for example, EpiInvert might even give negative $R_{t}$ estimates on some occasions), or makes $R_{t}$ hard to estimate with high precision, making it difficult to assess whether intervention measures in place in the community to mitigate disease spread are unduly strict. Many of the countries and territories with long periods of successful mitigation, particularly in Asia [31,32], faced this issue during the first year and a half of the pandemic, complicating decision making. A fundamental issue is the inverse correlation between the reproduction number in successive time points—future cases being explainable if $R_{t}$ is high and $R_{t+1}$ low, or vice versa, or anywhere in between—and the target of inference being the marginal distribution for these quantities. Other approaches have tackled this using smoothing approaches to share information between nearby time points, such as EpiFilter [6,9] which utilises Bayesian recursive filters to good effect by introducing a Gaussian relationship between neighbouring $R_{t}$s. 

This study proposes an alternative means of pooling information across time, by linking the estimation of the ensemble {$R_{t}$} to time-varying covariates whose effect may potentially be preserved across prolonged periods of the epidemic, and with a feasible relationship—at least correlative—with transmission rates. This approach performed no worse than three other, prominent methods, EpiEstim [3,8], EpiFilter [6,9], and EpiInvert [7], and in some situations compared favourably, particularly when incidence was dominated by imported cases and quarantine measures for returnees had taken effect. Furthermore, the smoothing techniques in the existed methods, though successfully reducing uncertainty in low incidence scenarios, tend to keep the estimates far below unity and make it impossible to respond to sudden changes in the transmissibility, which might not be reflected in the number of reported cases, especially if daily case counts are too small. Therefore, the relatively large uncertainty in $R_{t}$ estimates by EpiRegress compared to that by EpiEstim or EpiFilter, might not necessarily be a drawback, as was demonstrated in the simulation results. The approach additionally lends itself well to nowcasting the effective reproduction rate when future cases due to the current cases are yet to emerge but the time-varying covariates can be measured in near real-time. This is not the case for some important variables we considered, such as mobility data which were made public only after a lag [33], but other data streams without this restriction may be possible for governments with modern surveillance systems, such as the Republic of Korea which deployed big data capture to good effect from an early stage of the COVID-19 pandemic [32,34]. Such nowcasting of $R_{t}$ would help policy makers respond rapidly to any upsurge in risks. 

Naively, we might hope that the inclusion of covariates that are related to $R_{t}\;,$ through a regression framework, would permit inferences on the key factors associated with growing transmissibility, as Beest et al., did when they estimated impacts of several influenza-related factors [35]. Such inference is, unfortunately, prevented by the high collinearity between various mobility, epidemiological, and policy covariates involved in EpiRegress, which frequently move in tandem as multiple policy changes or behavioural changes conterminously vary. As a result, it is unlikely that the impact of specific policies can be obtained through our approach (though an approach akin to a meta-analysis over many countries might permit such associations to be derived [36]). While this may be seen as a weakness, it also points to the robustness of the methodology to model misspecification, for even if more distal covariates are included instead of those more proximately related to transmission, the inferred $R_{t}$, the key estimand of interest, is little changed.

Another advantage of our approach is that it explicitly distinguishes imported cases from autochthonous ones. The effect of the COVID-19 pandemic on international, and in some cases intranational, travel has been unprecedented [37,38]. Differences in quarantine policies in different polities has led to marked variability in the importance of imported cases to the local epidemic, with countries such as China, New Zealand, and Singapore operating very successful quarantine systems [2,39]. With little local infection and a quarantine system that leads to little leakage, imported cases contribute little to secondary spread, but counts of imported cases are often not differentiated from autochthonous ones in international databases. As a result, estimates of $R_{t}$ that do not distinguish these case types give a misleading depiction of the effectiveness of control in the country receiving infected international travellers. Future efforts to standardise data reporting should seek to explicitly distinguish these two groups for this reason. In our analysis, we found that assuming the same transmission potential of imported and local cases, or excluding the former altogether, led to noticeable differences in the estimates using existing methods; neither approach was necessary in our framework, however.

Limitations of this study include the assumption that all serial intervals are constant for all the regions explored. Differences in reporting times between linked cases in Singaporean households may be shorter than those between linked cases not sharing the same living space, causing an underestimate of $R_{t}$. The diverse nonpharmaceutical interventions taken by different regions or by one region at different time periods may also cause fluctuations in the serial interval distribution [40]. The $R_{t}$ estimates under EpiRegress displayed a weekly cycle which we attribute to the inclusion of Google mobility data, which is an important factor in the estimates, but these weekend dips may not truly reflect changes in risk [41]. Furthermore, to get better estimate of $R_{t}$s for each region, we preferred not to exclude any of the variables listed in either mobility data or policy data, despite the existence of collinearities [42]. As aforementioned, this did not deleteriously affect estimates of $R_{t}$ but did prohibit us from assessing their individual impacts on $R_{t}$. Lastly, we also assumed a constant under-reporting rate which may be dependent on testing practices [3] and that the lag between daily case counts and response to interventions was negligible, but we explored the latter in the Supplementary Information which suggests that the estimates of $R_{t}$ were robust to this in all four regions (Table S9). 

Despite these limitations, we believe that the extension of methods to estimate the effective reproduction number that account for time-varying covariates that are plausibly linked to transmission potential using our framework provides a useful addition to our analytic armamentarium for future outbreaks. It will be particularly valuable for places and times when outbreaks are smaller, in small countries or subnational regions, or when mitigation measures remain effective. Although we applied it to COVID-19, it will be applicable to other infectious diseases causing explosive outbreaks, when data on both cases and exogeneous factors are available.

## Figures and Tables

**Table 1:** Comparison of mean absolute error (MAE) and mean absolute percentage error (MAPE) of $R_{t}$ point estimates by EpiRegress, EpiEstim, EpiFilter, and EpiInvert with imported cases included (‘import’) or excluded (‘local’) in the total case counts, as well as the successful coverage rate (SCR) of the estimated 95% CrIs by EpiRegress.

		Scenario 1				Scenario 2			
Simulation		1	2	3	4	1	2	3	4
**MAE**	**EpiRegress**	0.19	0.25	0.24	0.09	0.33	0.43	0.35	0.29
	**EpiEstim**	0.92	0.60	0.85	0.41	0.80	0.95	0.90	0.75
	**EpiFilter**	0.88	0.59	0.84	0.38	0.79	0.93	0.92	0.73
	**EpiInvert (Local)**	0.24	0.33	NA	0.24	0.42	20	0.67	0.88
	**EpiInvert (Import)**	0.62	0.39	NA	0.25	0.62	0.45	0.66	0.59
**MAPE**	**EpiRegress**	0.15	0.18	0.22	166	0.25	0.26	0.27	0.26
	**EpiEstim**	0.81	0.63	0.69	2596	0.75	0.61	0.66	0.64
	**EpiFilter**	0.75	0.57	0.63	2699	0.69	0.59	0.68	0.56
	**EpiInvert (Local)**	2.07	0.29	NA	2608	0.77	25	0.87	1.1
	**EpiInvert (Import)**	1.78	1.16	NA	2595	0.83	0.64	0.83	0.68
**SCR (%)**	**EpiRegress**	91	98	94	99	97	97	97	96
	**EpiEstim**	21	38	18	35	29	23	34	49
	**EpiFilter**	18	20	12	34	14	12	14	22

## Data Availability

Code and data are available at https://github.com/ShihuiJin/EpiRegress (accessed on 31 May 2022).

## Supplementary Materials

The following supporting information can be downloaded at: https://www.mdpi.com/article/10.3390/v14071576/s1, Table S1: Descriptions and interpretations of different scores for different indicators in OxCGRT. Table S2: List of factors included in the full and hybrid (subset) models. Table S3: Squared root of the mean of squared distance from real case counts in the testing sets to the corresponding mean and boundaries of the 50% and 95% credible intervals of the posterior distributions of the number of reported local case counts. Table S4: Fit of the model: Percentage of the estimated 50% and 95% credible intervals (CrI) for local cases that successfully covered the observations. Table S5: Forecasting accuracy for different number of days after the end of the inference window. Table S6: Deviance Information Criterion (DIC) of models. Table S7: Mean absolute difference between posterior median of *R*_t_ estimates given by the model including all the available factors. Table S8: Smoothness (average absolute difference between neighbouring values) of posterior median of *R*_t_ estimates given by the model. Table S9: Forecasting accuracy when allowing different times of delays in report for the four regions. Figure S1: Visualization of Google mobility data. Figure S2: Numbers of daily COVID-19 imported and local (community) cases from 1 January to 30 September 2021. Figure S3: Log-normal approximation of the empirical serial interval distribution from 157 pairs of household infections in Singapore. Figure S4: Comparison of *R*_t_ estimates by EpiRegress, EpiEstim, EpiFilter and EpiInvert against the true values for the four different incidence curves in scenario 1. Figure S5: Comparison of *R*_t_ estimates by EpiRegress, EpiEstim, EpiFilter and EpiInvert against the true values for the four different incidence curves in scenario 1. Figure S6: Comparison of the reported and estimated local case counts. Figure S7: Comparison of point estimates of *R*_t_ based on three different regression models. Figure S8: Comparison of estimates in scenarios when phase factors were or were not considered as part of the ***X*** covariates for Singapore. Figure S9: comparison of *R*_t_ estimates in scenarios when dormitory cases were or were not regarded as part of the imported case counts for Singapore.
