# Supplementary material for: EpiRegress: A Method to Estimate and Predict the Time-Varying Effective Reproduction Number
Source: Viruses. 2022 Jul 20;14(7):1576. doi: 10.3390/v14071576 (PMC9323923; doi:10.3390/v14071576)
Supplement: Supplementary file 1 [file viruses-14-01576-s001.zip › viruses-1775505-supplementary.pdf]

Supplementary Materials

Table S1. Descriptions and interpretations of different scores for different indicators in OxCGRT.

Category	Name	Descriptions	Instructions of the Scores
**Closures and containment**	School closing	Record closings of schools and universities	0: no measures<br>1: recommend closing, or all schools open with alterations resulting in significant differences compared to usual, non-Covid-19 operations<br>2: require closing only some levels or categories<br>3: require closing all levels
	Workplace closing	Record closings of workplaces	0: no measures<br>1: recommend closing<br>2: require closing for some sectors or categories of workers<br>3: require closing all-but-essential workplaces
	Cancel public events	Record cancelling public events	0: no measures<br>1: recommend cancelling<br>2: require cancelling
	Restrictions on gatherings	Record the cut-off size for bans on gatherings	0: no restrictions<br>1: restrictions on very large gatherings (above 1000 people)<br>2: restrictions on gatherings between 101-1000 people<br>3: restrictions on gatherings between 11-100 people<br>4: restrictions on gatherings of 10
	Close public transport	Record closing of public transport	0: no measures<br>1: recommend closing or significantly reduce volume/route/means of transport available<br>2: require closing or prohibit most citizens from using it
	Stay at home requirements	Record orders to "shelter-in- place" and otherwise confined to home.	0: no measures<br>1: recommend not leaving house<br>2: require not leaving house with exceptions for daily exercise, grocery shopping, and 'essential' trips<br>3: require not leaving house with minimal exceptions
	Movement restrictions	Record restrictions on internal movement	0: no measures<br>1: recommend not to travel between regions/cities<br>2: internal movement restrictions in place
	International travel	Record restrictions on international travel	0: no measures<br>1: Screening<br>2: Quarantine arrivals from high-risk regions<br>3: Ban on arrivals from some regions 4 – Ban on all regions or total border closure
**Economic Measures**	Income support	Record if the government is covering the	0: no income support<br>1: government is replacing less than 50% of lost salary (or if a flat sum, it is less than 50% median

		salaries or providing direct cash payments, universal basic income, or similar, of people who lose their jobs or cannot work.	salary)<br>2: government is replacing 50% or more of lost salary (or if a flat sum, it is greater than 50% median salary)
	Debt relief	Record if government is freezing financial obligations	0: no<br>1: narrow relief, specific to one kind of contract<br>2: broad debt/contract relief
**Health Measures**	Public information campaigns	Record presence of public information campaigns	0: no COVID-19 public information campaign<br>1: public officials urging caution about COVID-19<br>2: coordinated public information campaign
	Testing policy	Who can get tested?	0: no testing policy<br>1: only those who both (a) have symptoms and (b) meet specific criteria<br>2: testing of anyone showing COVID-19 symptoms<br>3: open public testing
	Contact tracing	Are governments doing contact tracing?	0: no contact tracing<br>1: limited contact tracing - not done for all cases<br>2: comprehensive contact tracing - done for all identified cases
	Facial Coverings	Record policies on the use of facial coverings outside the home	0: no policy<br>1: recommended<br>2: required in some specified public spaces outside the home with other people present, or some situations when social distancing not possible<br>3: required in all public spaces outside the home with other people present or all situations when social distancing not possible<br>4: required outside the home at all times regardless of location or presence of other people
	Vaccination Policy	Record policies for vaccine delivery for different groups	0: no availability<br>1: availability for one of following: key workers/ clinically vulnerable groups / elderly groups<br>2: availability for two of following: key workers/ clinically vulnerable groups / elderly groups<br>3: availability for all of following: key workers/ clinically vulnerable groups / elderly groups<br>4: availability for all three plus partial additional availability<br>5: universal availability
	Protection of elderly people	Record policies for protecting elderly people in	0: no measures<br>1: recommended isolation, hygiene, and visitor restriction measures in LTCFs and/or elderly

	Long Term Care Facilities (LTCF) and/or the community and home setting	people to stay at home<br>2: narrow restrictions for isolation, hygiene in LTCFs, some limitations on external visitors and/or restrictions protecting elderly people at home<br>3: extensive restrictions for isolation and hygiene in LTCFs, all non-essential external visitors prohibited, and/or all elderly people required to stay at home and not leave the home with minimal exceptions, and receive no external visitors

Policy indices are calculated directly from scores of different combinations of the individual component indicators. The stringency index is comprised of all the indicators in the category of ‘closures and containment’ together with ‘public information campaigns’. The government response index makes use of all the indicators listed above. The containment health index is from the indicators in the category of ‘closures and containment’ and ‘healthcare measures’, whilst the economic support index only utilises the two indicators in the category of ‘economic measures’.

Table S2. List of factors included in the full and hybrid (subset) models.

**Mobility factors**		**Epidemiological factors**		**Policy factors**	
**Full**	**Hybrid**	**Full**	**Hybrid**	**Full**	**Hybrid**
• Residential<br>• Workplace<br>• Retail & recreation<br>• Grocery and pharmacy<br>• Parks<br>• Transit stations	• Residential<br>• Retail & recreation	• Vaccination rate<br>• Delta Variant proportion	• Vaccination rate	• Phase information (for Singapore only)<br>• Stringency index<br>• Government response index<br>• Containment health index<br>• Economic support index<br>• School closing<br>• Workplace closing<br>• Cancel public events<br>• Restrictions on gatherings<br>• Close public transport<br>• Stay at home requirements<br>• Movement restrictions<br>• International travel<br>• Income support<br>• Debt relief<br>• Public information campaigns<br>• Testing policy<br>• Contact tracing<br>• Facial Coverings<br>• Vaccination Policy<br>• Protection of elderly people	• School closing<br>• Workplace closing<br>• Cancel public events<br>• Restrictions on gatherings<br>• Close public transport<br>• Stay at home requirements<br>• Movement restrictions<br>• International travel<br>• Income support<br>• Debt relief<br>• Public information campaigns<br>• Contact tracing<br>• Facial Coverings<br>• Protection of elderly people

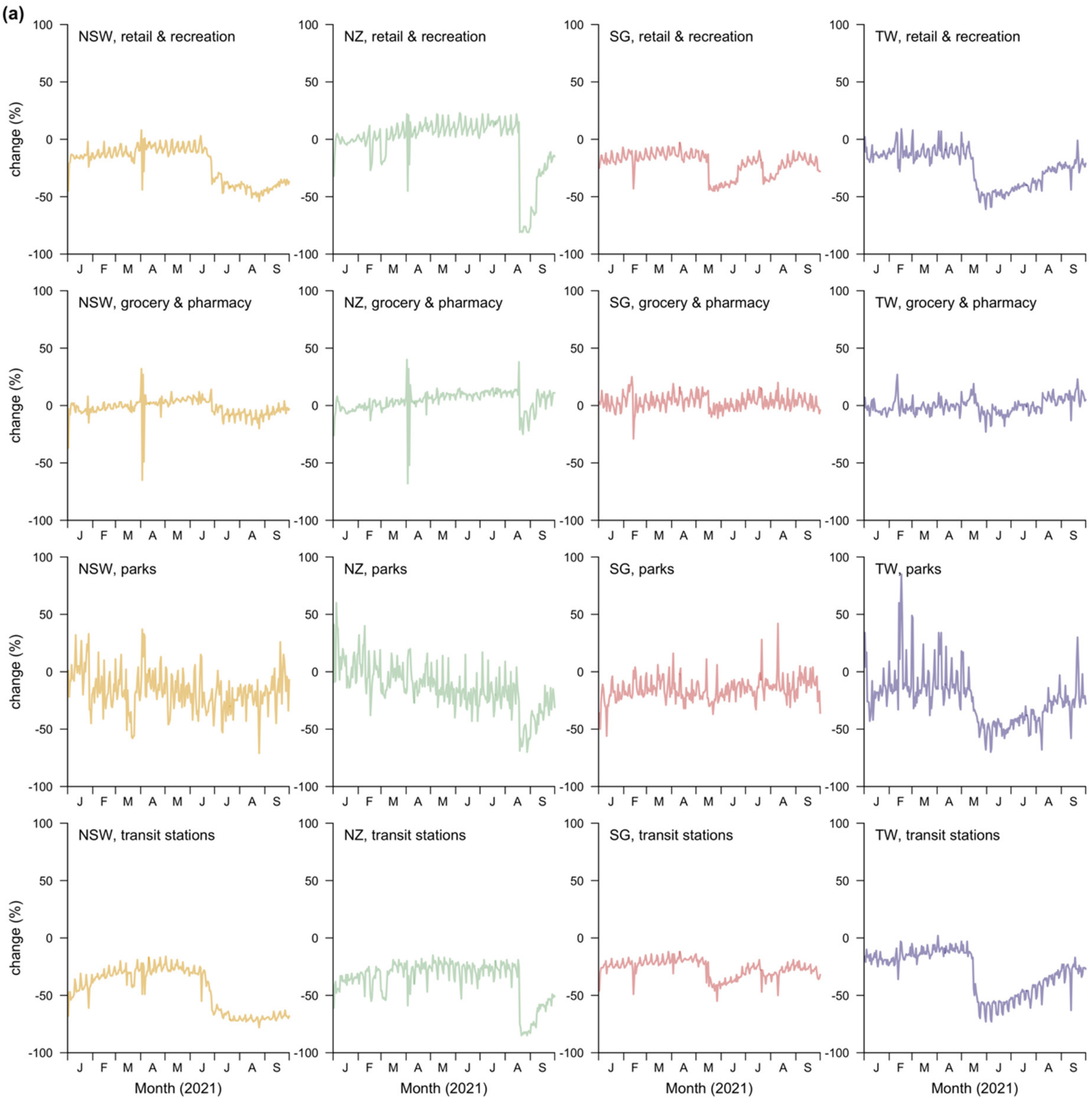
(a)
NSW, retail & recreation
NZ, retail & recreation
SG, retail & recreation
TW, retail & recreation
NSW, grocery & pharmacy
NZ, grocery & pharmacy
SG, grocery & pharmacy
TW, grocery & pharmacy
NSW, parks
NZ, parks
SG, parks
TW, parks
NSW, transit stations
NZ, transit stations
SG, transit stations
TW, transit stations
change (%)
100
50
0
-50
-100
J F M A M J J A S
Month (2021)

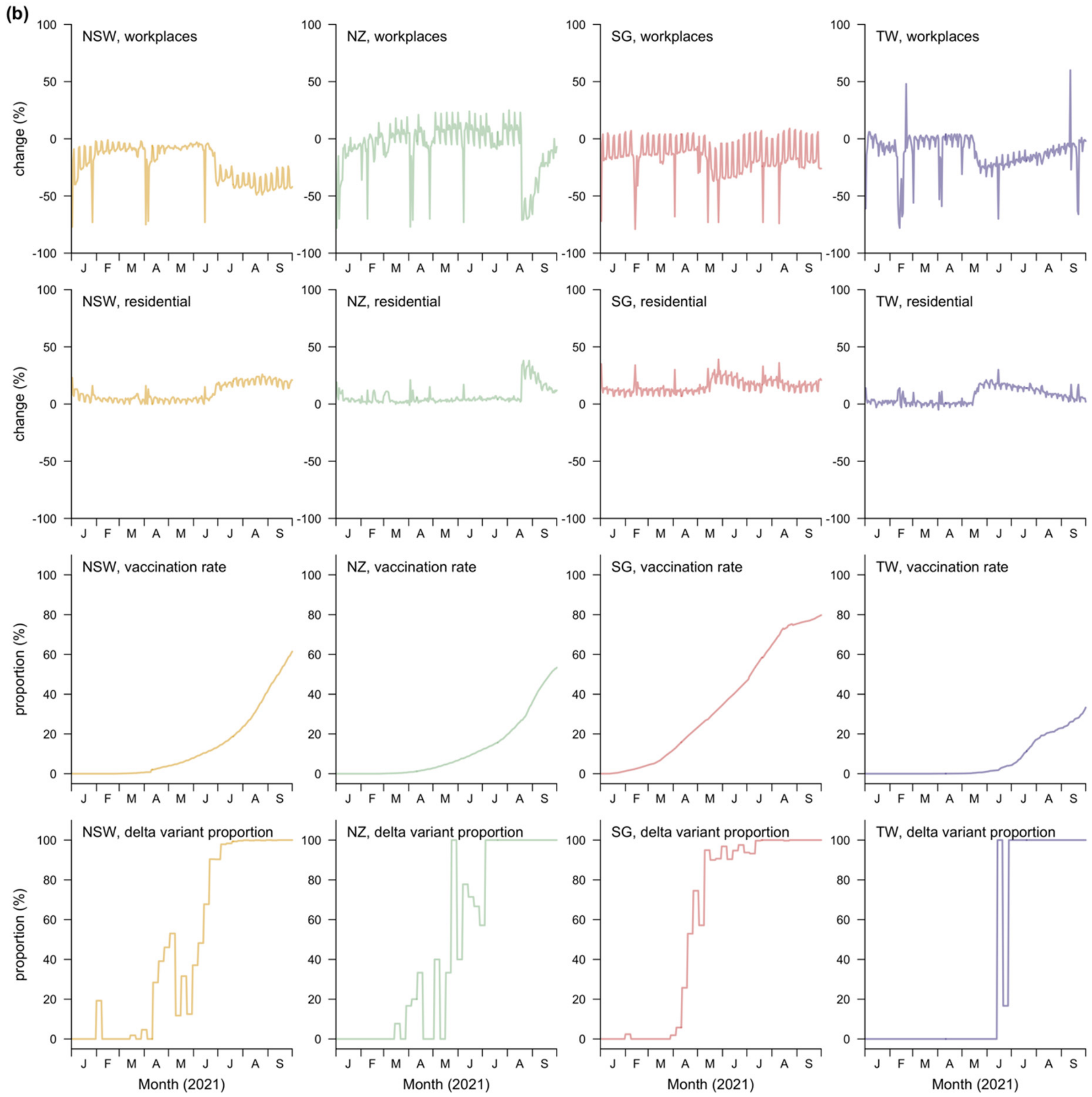
(b)
NSW, workplaces
NZ, workplaces
SG, workplaces
TW, workplaces
NSW, residential
NZ, residential
SG, residential
TW, residential
NSW, vaccination rate
NZ, vaccination rate
SG, vaccination rate
TW, vaccination rate
NSW, delta variant proportion
NZ, delta variant proportion
SG, delta variant proportion
TW, delta variant proportion
change (%)
proportion (%)
J F M A M J J A S
Month (2021)

(c)

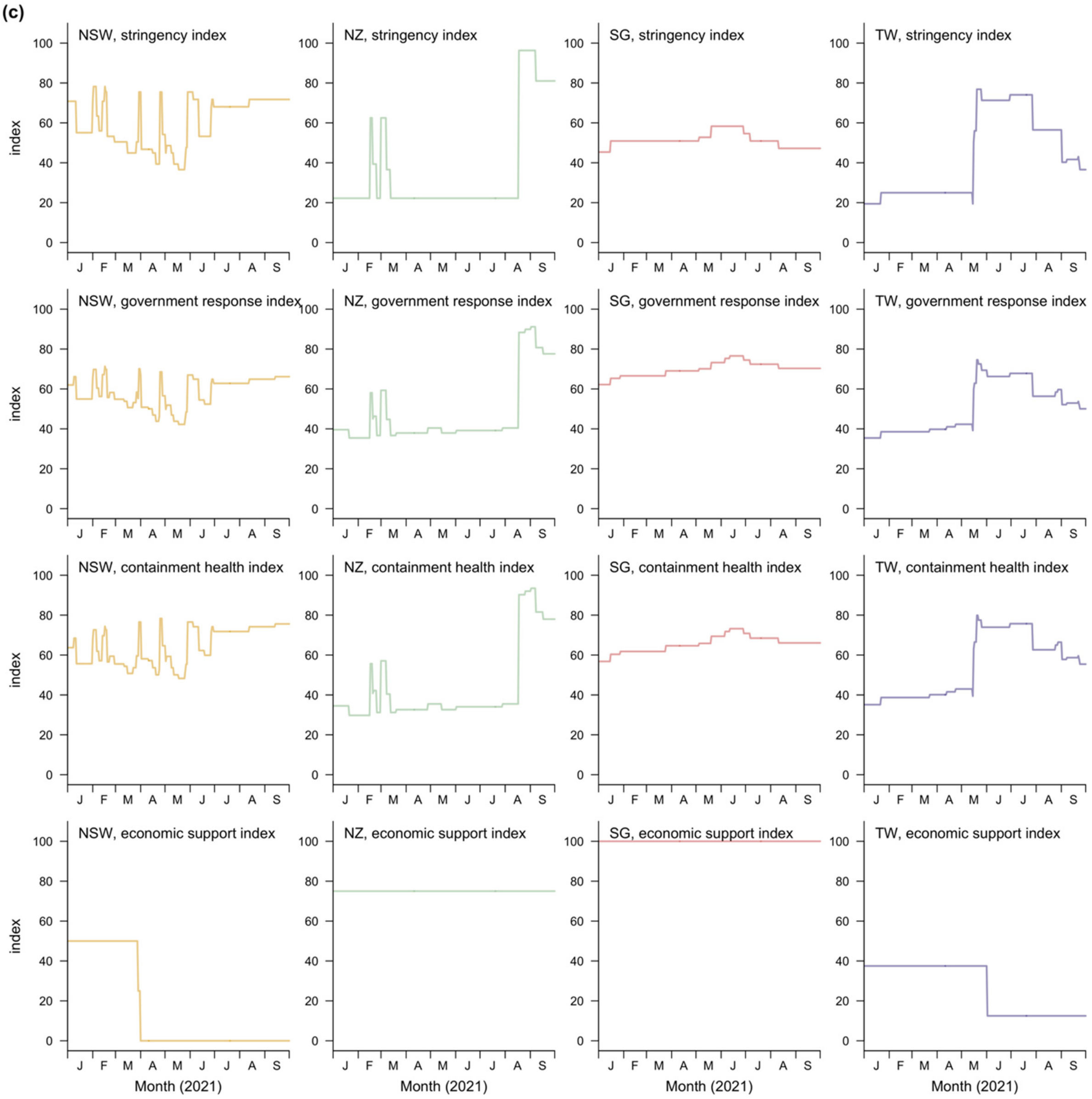
NSW, stringency index
NZ, stringency index
SG, stringency index
TW, stringency index
NSW, government response index
NZ, government response index
SG, government response index
TW, government response index
NSW, containment health index
NZ, containment health index
SG, containment health index
TW, containment health index
NSW, economic support index
NZ, economic support index
SG, economic support index
TW, economic support index
index
0
20
40
60
80
100
J F M A M J J A S
Month (2021)

(d)

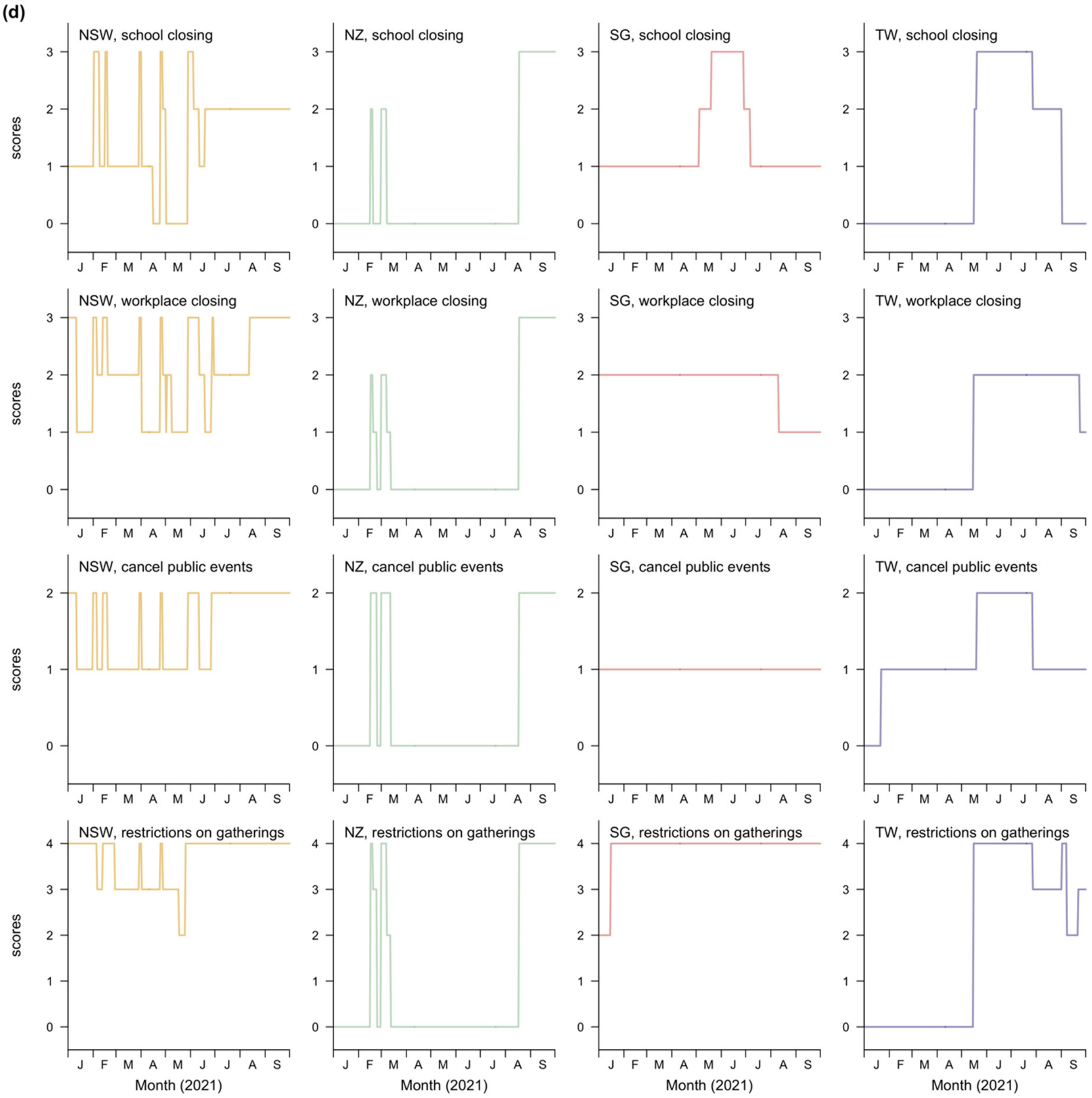

NSW, school closing
NZ, school closing
SG, school closing
TW, school closing
NSW, workplace closing
NZ, workplace closing
SG, workplace closing
TW, workplace closing
NSW, cancel public events
NZ, cancel public events
SG, cancel public events
TW, cancel public events
NSW, restrictions on gatherings
NZ, restrictions on gatherings
SG, restrictions on gatherings
TW, restrictions on gatherings
scores
J F M A M J J A S
Month (2021)

(e)

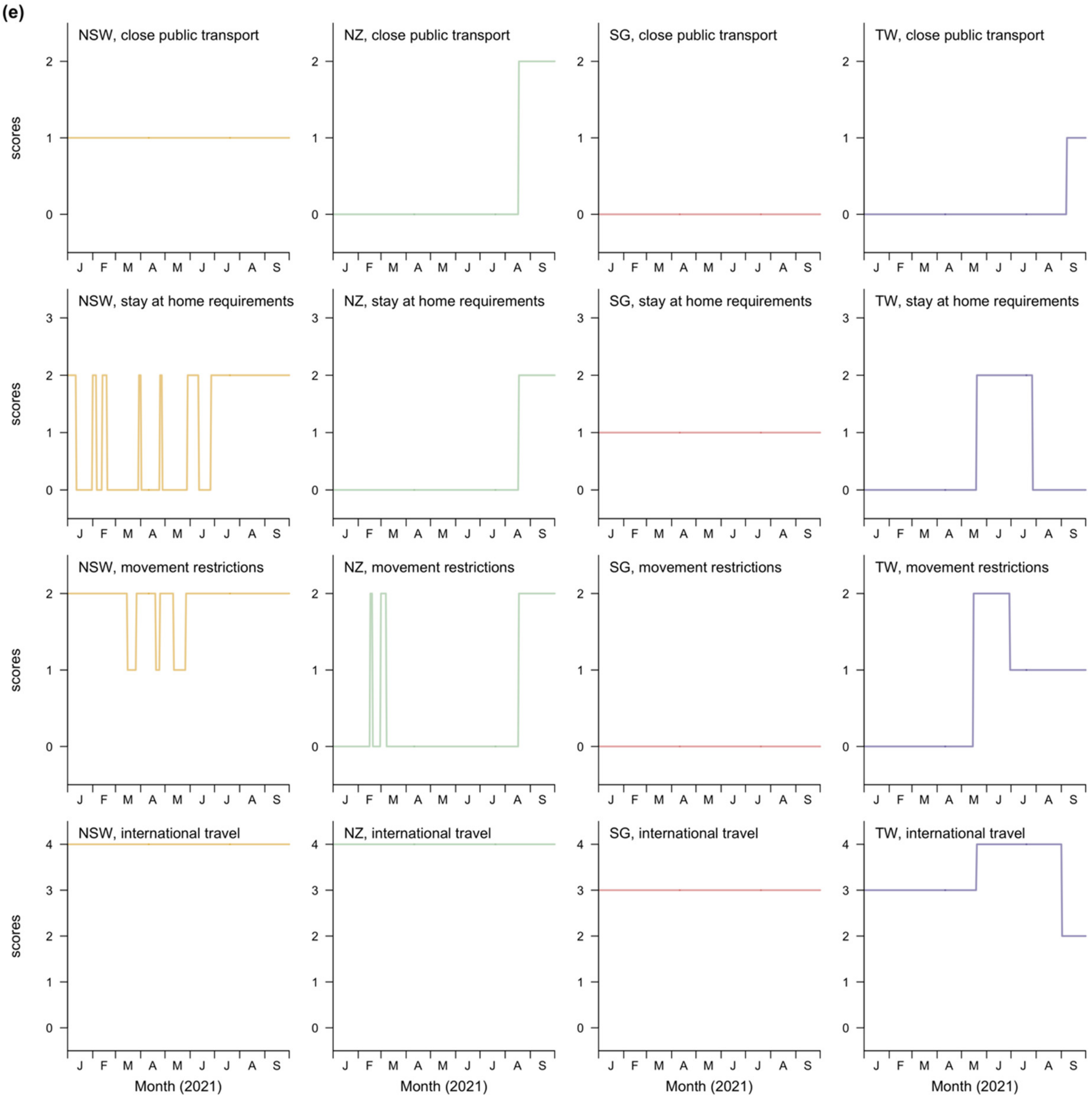
NSW, close public transport
NZ, close public transport
SG, close public transport
TW, close public transport
NSW, stay at home requirements
NZ, stay at home requirements
SG, stay at home requirements
TW, stay at home requirements
NSW, movement restrictions
NZ, movement restrictions
SG, movement restrictions
TW, movement restrictions
NSW, international travel
NZ, international travel
SG, international travel
TW, international travel
scores
J F M A M J J A S
Month (2021)

(f)

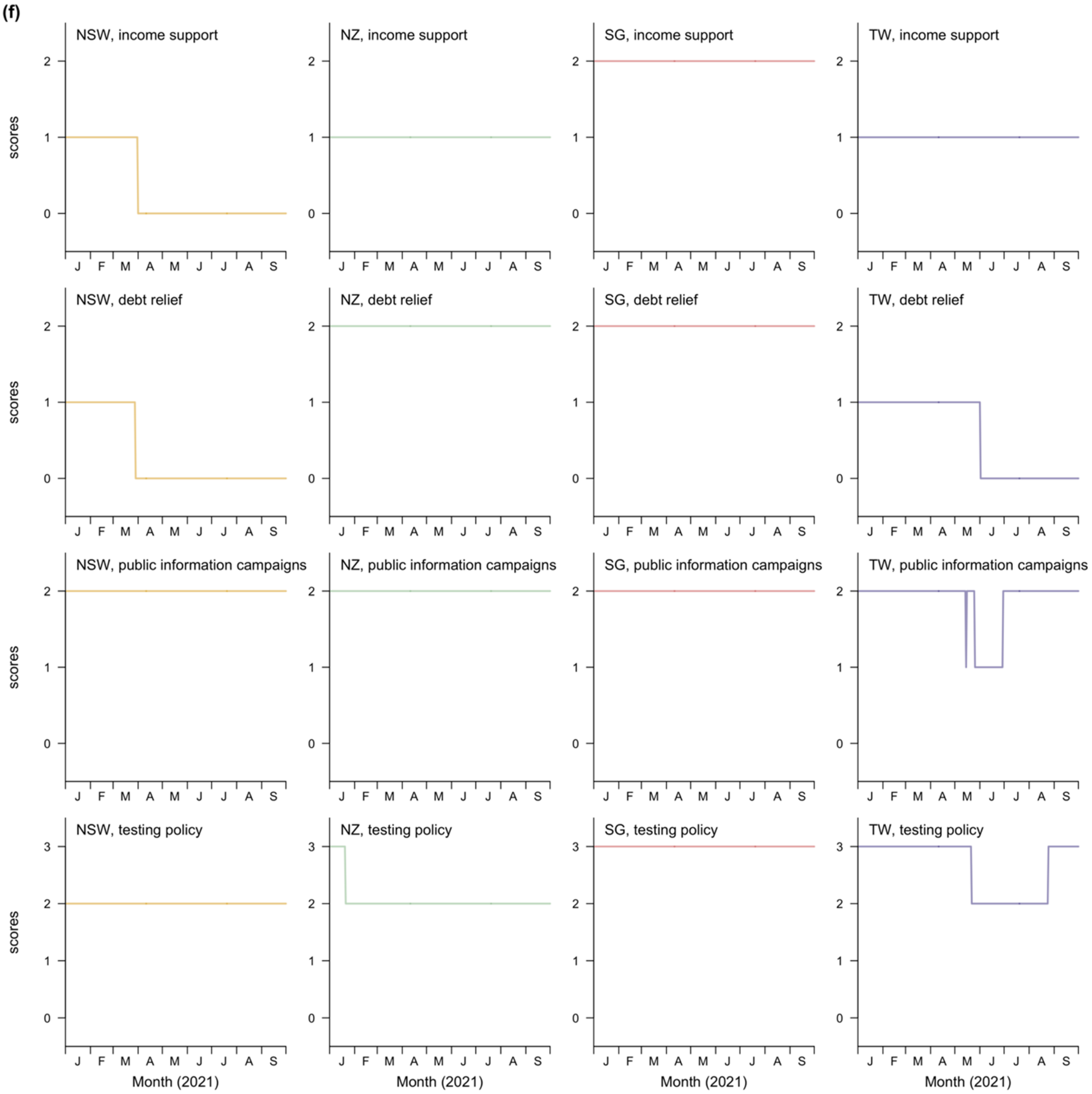
NSW, income support
NZ, income support
SG, income support
TW, income support
NSW, debt relief
NZ, debt relief
SG, debt relief
TW, debt relief
NSW, public information campaigns
NZ, public information campaigns
SG, public information campaigns
TW, public information campaigns
NSW, testing policy
NZ, testing policy
SG, testing policy
TW, testing policy
scores
Month (2021)
J F M A M J J A S

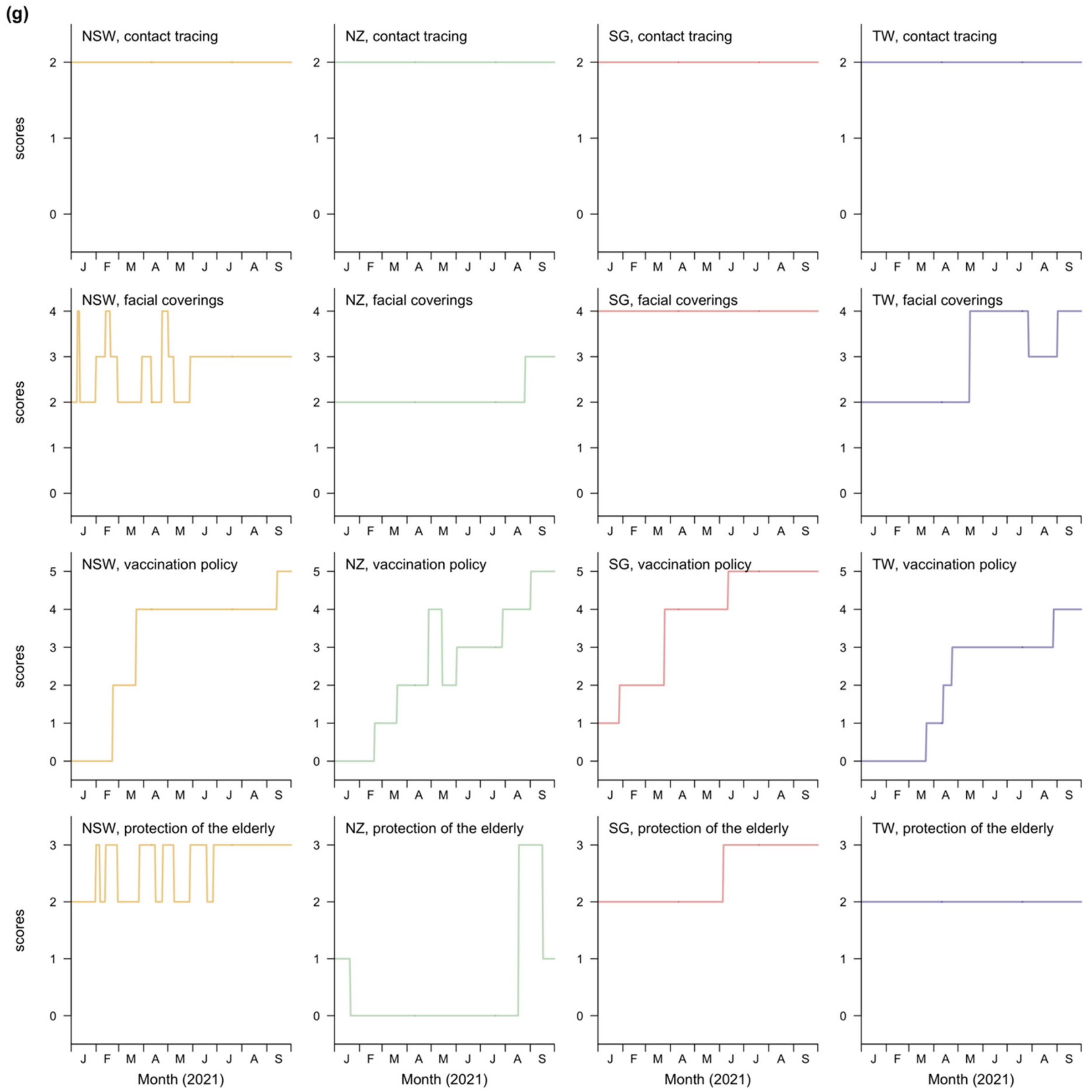

Figure S1. Visualization of Google mobility data (subplot (**a**) and the first two rows in subplot (**b**)), epidemiological data (vaccination rates and delta variant proportions, the second two rows in subplot (**b**)) and OxCGRT policy data (subplot (**c**)–(**g**)) from 1 January to 30 September 2021 in: (i) New South Wales, Australia; (ii) New Zealand; (iii) Singapore; (iv) Taiwan, China.

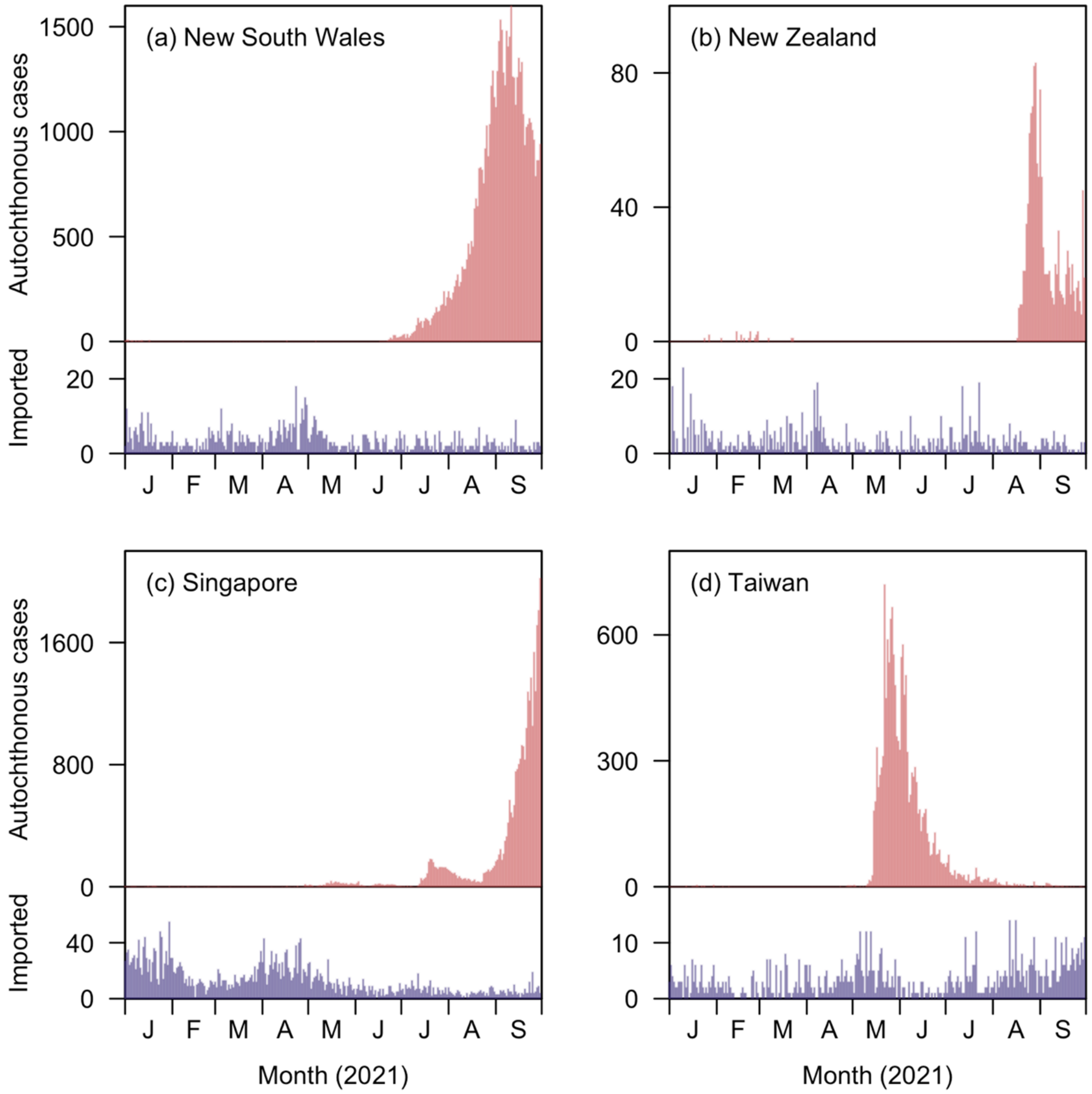

Figure S2. Numbers of daily COVID-19 imported and local (community) cases from 1 January to 30 September 2021 in: (**a**) New South Wales, Australia; (**b**) New Zealand; (**c**) Singapore; (**d**) Taiwan, China.

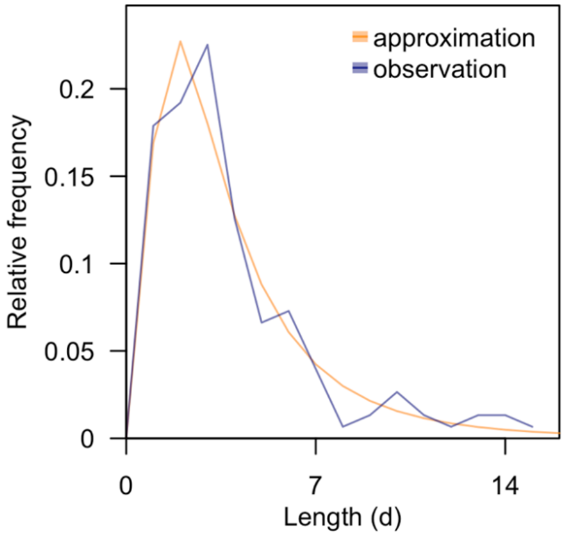

Figure S3. Log-normal approximation of the empirical serial interval distribution from 157 pairs of household infections in Singapore.

Selection of the Bayesian Lasso Parameter, λ

We utilised Monte Carlo cross-validation to choose which λ to use in the model. We created 50 random split of the 273 daily case counts in 2021, with 25 in the testing set and the rest in the training set. Case counts in the testing set were not involved in the likelihood calculation. We let λ equal to $e^i, i = -3, -2.5, -2, \cdots 3,3.5,4$ and compared the real case counts with the corresponding means and the boundaries of 50% and 95% credible intervals of the posterior predictive distributions. A λ of 5 was used being very similar to the results of λ of 4.5.

Table S3. Squared root of the mean of squared distance from real case counts in the testing sets to the corresponding mean and boundaries of the 50% and 95% credible intervals of the posterior distributions of the number of reported local case counts.

λ	Distance to Mean	Distance to the 50% Credible Interval	Distance to the 95% Credible Interval
**0.05**	45	35	21
**0.08**	48	38	23
**0.14**	44	34	20
**0.22**	45	35	21
**0.37**	43	33	19
**0.61**	45	35	20
**1.0**	42	32	18
**1.6**	41	31	16
**2.7**	41	31	16
**4.5**	41	30	15
**7.4**	42	31	15
**12**	43	31	16
**20**	44	32	16
**33**	43	32	17
**55**	44	32	17

Comparison of R_t Estimation for Different Incidence Curves by Using Four Different Methods

Using the 8 simulated incidence curves, we estimated R_ts with four different methods, namely RpiRegress, EpiEstim, EpiFilter and EpiInvert respectively. For the first three methods in which it is possible to account for effects of imported cases, we included the imported case counts in the denominator for estimating the effective reproduction number When using EpiInvert for R_t estimation, however, we considered two different ways of calculating the case counts, one with imported case counts excluded completely and the other with imported cases included. Estimation results for the first three methods consist of point estimates (posterior medians) and 95% CrI's while for EpiInvert, we only used the point estimates in the R outputs to plot and compare with the simulated 'true' values.

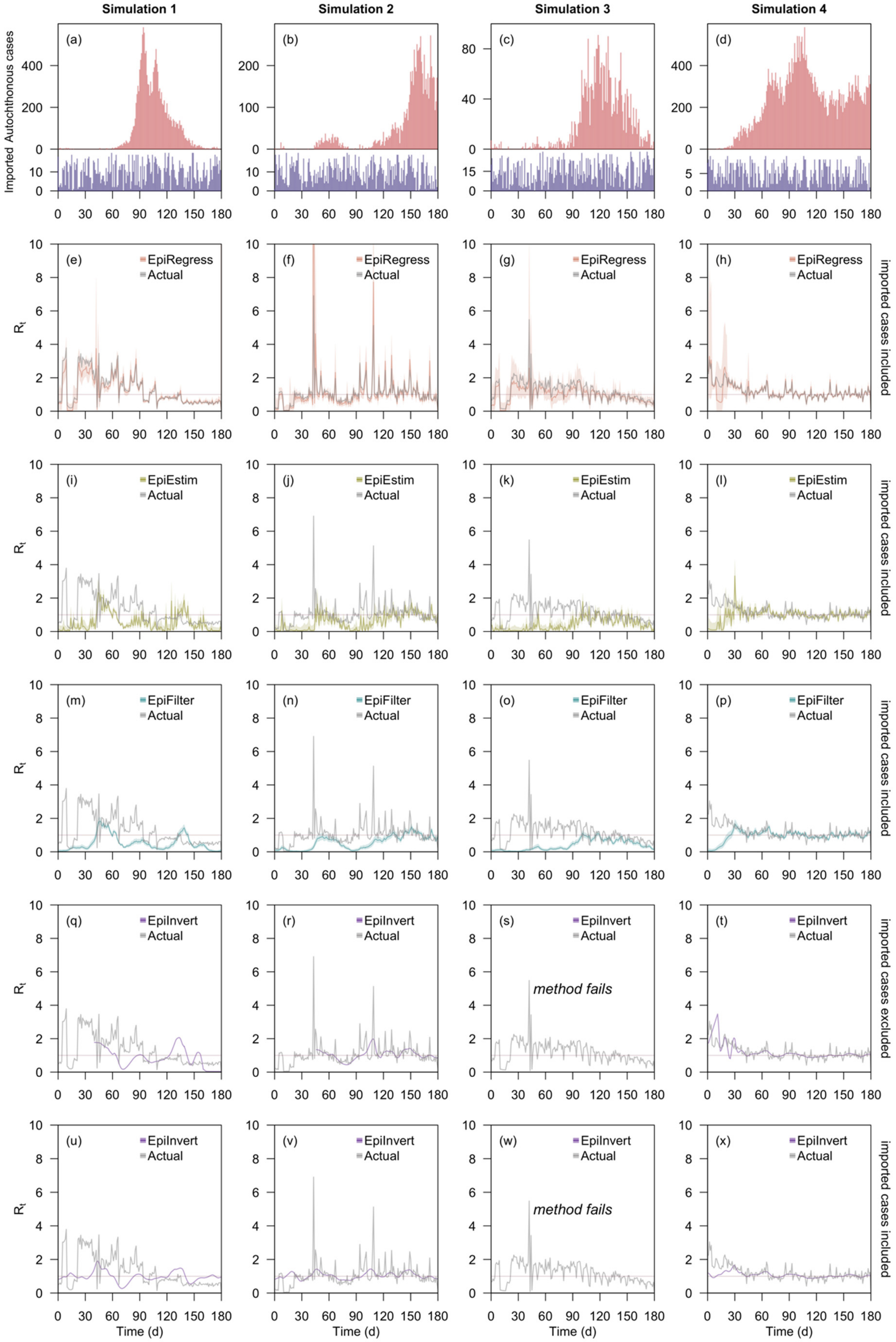

Figure S4. Comparison of R_t estimates by EpiRegress, EpiEstim, EpiFilter and EpiInvert against the true values for the four different incidence curves in scenario 1, where mobility, epidemiological and

policy data in New Zealand was utilized to form the $\boldsymbol{X}$ covariate matrix. EpiInvert with imported cases excluded in the total case counts failed to produce R_t point estimates for some time points. Additionally, for the third simulated incidence curve, where the maximum number of reported local cases in a day did not exceed 100 and there were many continuous days with zero or single-digit case counts, the method completely failed, no matter whether imported cases were included in the total case counts.

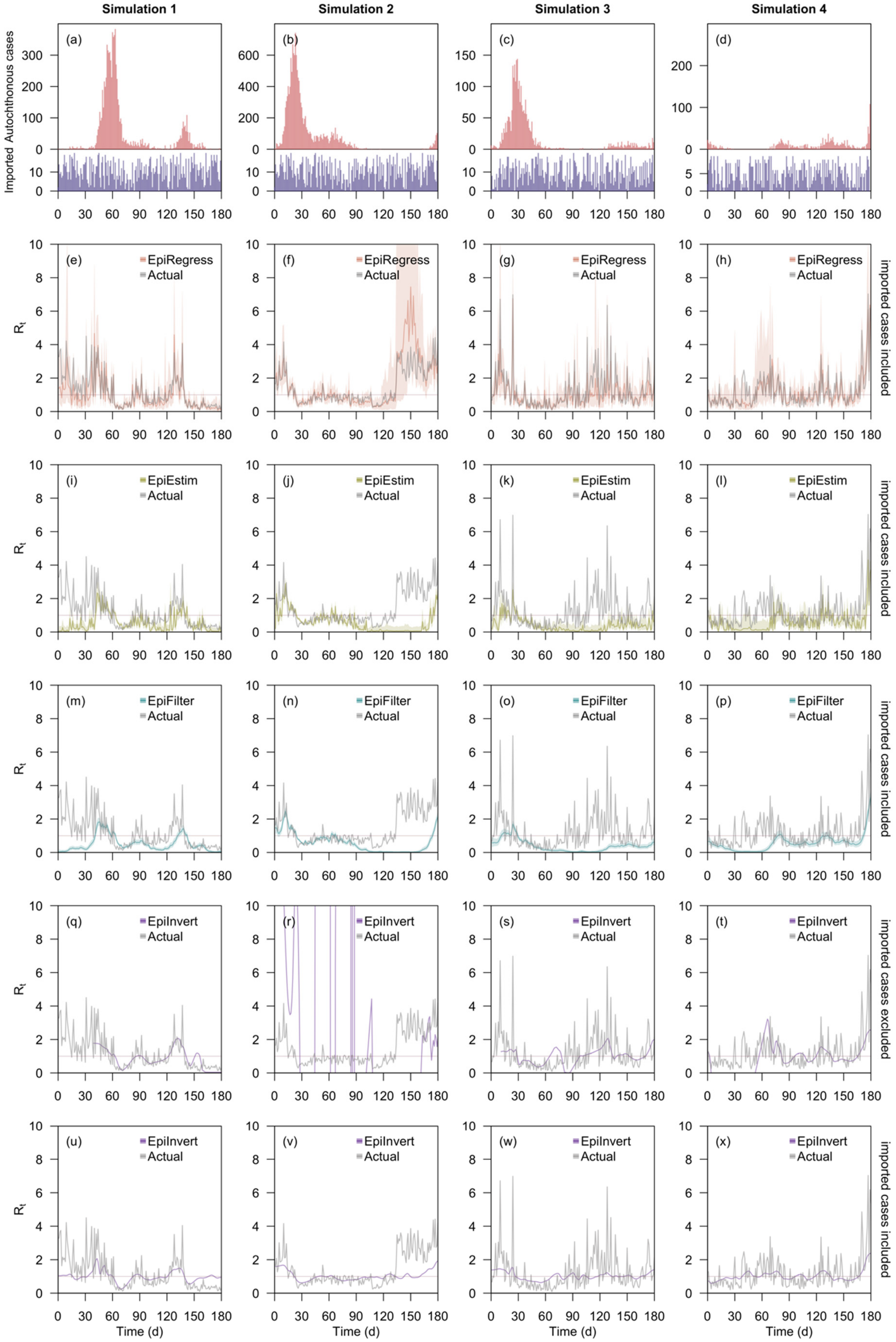

Figure S5. Comparison of R_t estimates by EpiRegress, EpiEstim, EpiFilter and EpiInvert against the true values for the four different incidence curves in scenario 1, where the $\boldsymbol{X}$ matrix consists of 20

randomly generated covariates, including 6 continuous variables and 14 ordinal ones with a range of 0–4. EpiInvert with imported cases excluded in the total case counts produced negative or even failed to produce R_t point estimates for some time points.

Comparison of Posterior Distributions of Case Counts with the Observed Values

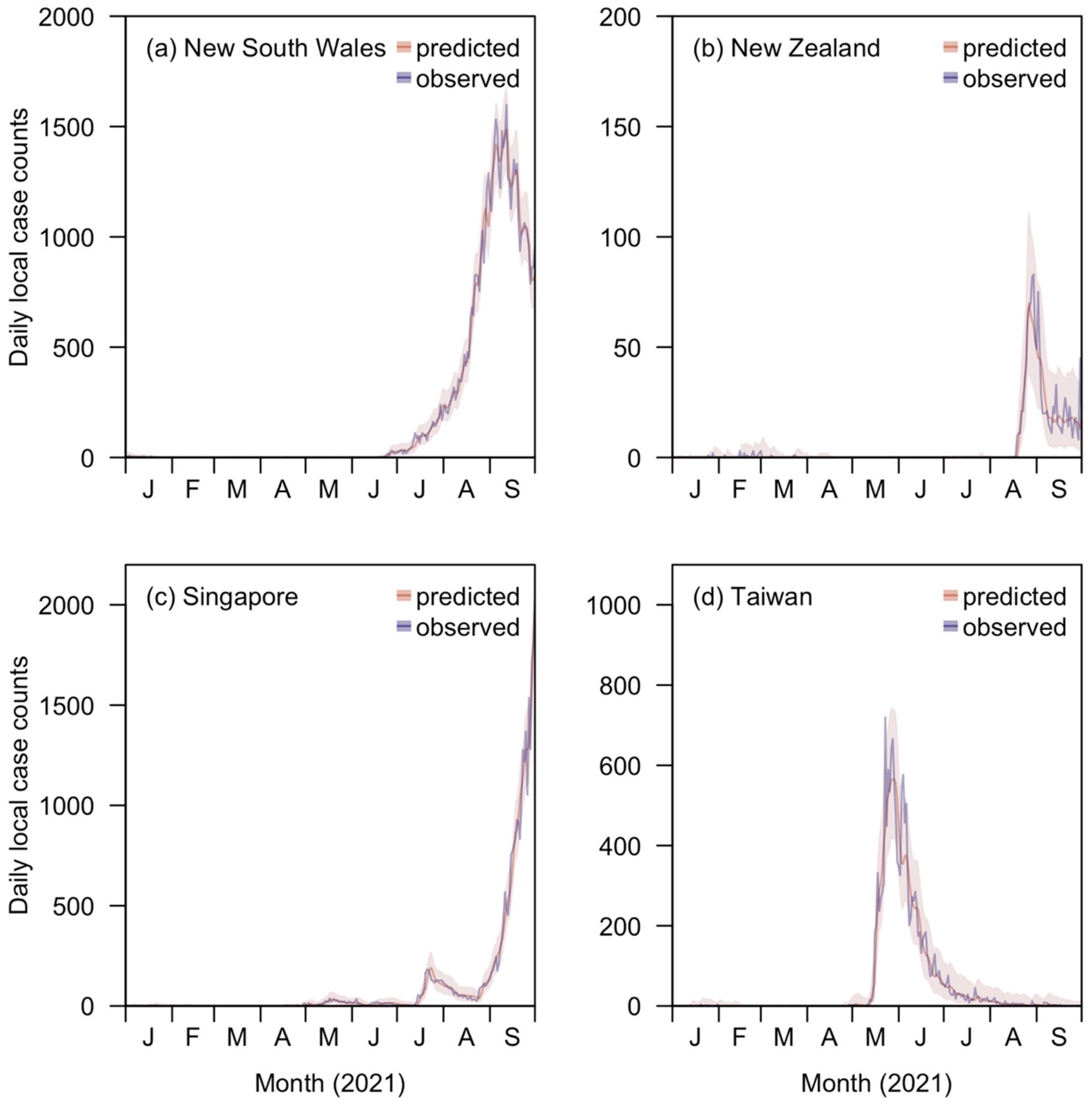

Figure S6. Comparison of the reported and estimated local case counts in: (**a**) New South Wales; (**b**) New Zealand ; (**c**) Singapore; (**d**) Taiwan, estimated by EpiRegress using all the covariates available (i.e. the full model) and a 9-month inference window from January to September 2021.

Table S4. Fit of the model: percentage of the estimated 50% and 95% credible intervals (CrI) for local cases that successfully covered the observations. The successful coverage rates might be large due to the existence of periods with virtually no reported cases for some regions.

Predictions in 50% CrI (NSW)	Predictions in 95% CrI (NSW)	Predictions in 50% CrI (NZ)	Predictions in 95% CrI (NZ)	Predictions in 50% CrI (SG)	Predictions in 95% CrI (SG)	Predictions in 50% CrI (TW)	Predictions in 95% CrI (TW)
79%	99%	90%	99%	70%	96%	78%	98%

Comparison of Forecasting Distributions of Case Counts with the Observed Values

We forecasted the next 7 days' case counts by using an inference window of 90 days for the four different regions. We performed the predictions for 240 different days (from 3 February to 30 September 2021), and calculated the proportion of observed values that fell in the 50% and 95% credible intervals of the posterior predictive distribution of the future case counts respectively (Table S5).

Table S5. Forecasting accuracy for different number of days after the end of the inference window (i.e. 'Forecasting day'). The successful coverage rates might be large due to the existence of periods with virtually no reported cases for some regions.

Forecasting day	Predictions in 50% CrI (NSW)	Predictions in 95% CrI (NSW)	Predictions in 50% CrI (NZ)	Predictions in 95% CrI (NZ)	Predictions in 50% CrI (SG)	Predictions in 95% CrI (SG)	Predictions in 50% CrI (TW)	Predictions in 95% CrI (TW)
**1**	71%	96%	86%	98%	63%	95%	80%	98%
**2**	70%	95%	88%	97%	61%	92%	78%	98%
**3**	70%	95%	87%	96%	62%	91%	77%	98%
**4**	69%	95%	88%	95%	60%	90%	79%	98%
**5**	71%	95%	85%	96%	60%	89%	80%	98%
**6**	71%	93%	86%	95%	62%	88%	79%	98%
**7**	70%	93%	84%	96%	58%	88%	79%	99%

Comparison of R_t Estimation for All the Four Regions by EpiRegress When Different Model Variants Were Used

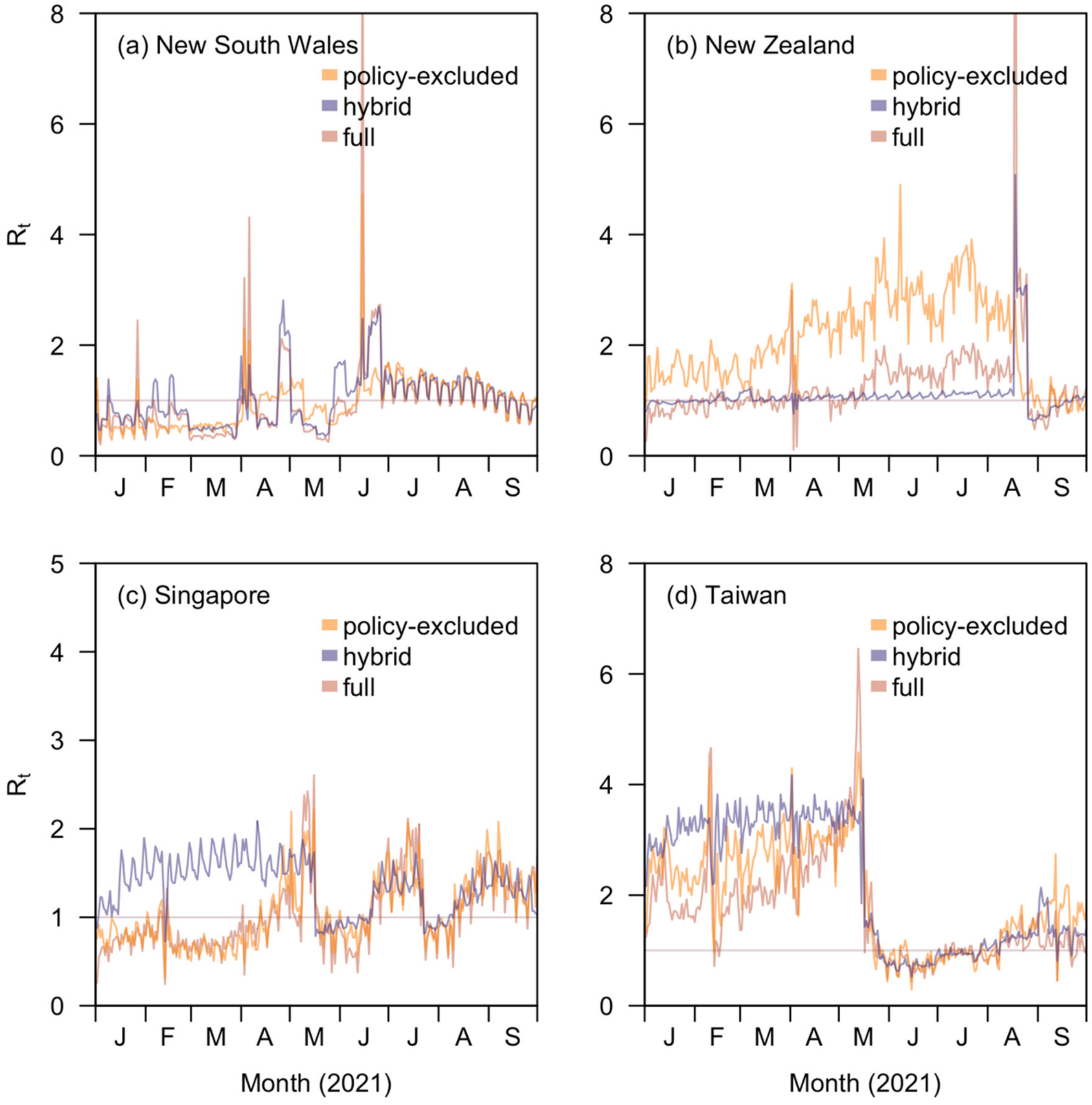

Figure S7. Comparison of point estimates of R_t based on three different regression models: (i) the full model with all covariates (red), (ii) the policy-excluded model without any policy-related covariates, and (iii) the hybrid model with fewer variables including 'retail and recreation' and 'residential' from google mobility data, and vaccination rate and all the indicator variables but 'testing policy' and 'vaccination policy' in the Oxford policy data (blue) in (**a**) New South Wales; (**b**) New Zealand; (**c**) Singapore; (**d**) Taiwan.

Comparison of R_t Estimation for Singapore by Either Including or Excluding Phase Information in the Covariates

We excluded factors relating to different phases in Singapore and redid the regression to estimate R_t's with EpiRegress when comparing the $\boldsymbol{\beta}$ coeffiecients of the covariates for different regions. Such exclusion did not significantly change both the posterior median and the 95% credible intervals of the R_t estimates for Singapore and the DICs for the two models were almost the same.

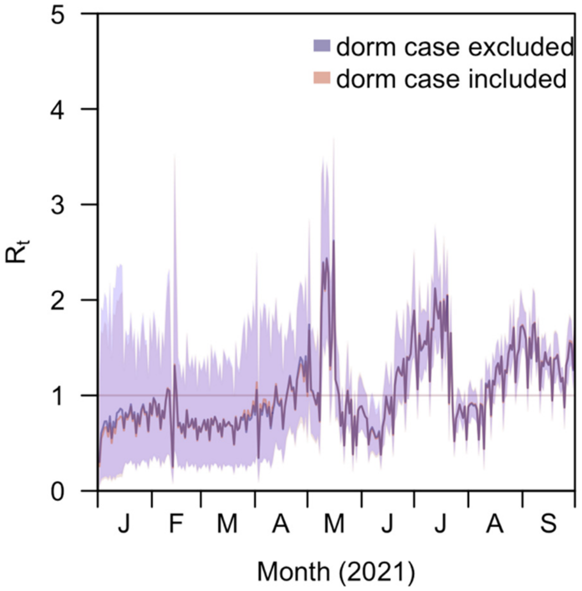

Figure S8. Comparison of estimates in scenarios when phase factors were or were not considered as part of the $\boldsymbol{X}$ covariates for Singapore.

Comparison of R_t Estimation When Either Including or Excluding Dormitory Cases as Imported Cases

Considering that dormitory residents were isolated in Singapore, we excluded dormitory cases in the local case counts. To test if they did affect the local cases, we modelled a scenario where dormitory cases were part of the imported cases and compared this with the original result, The two versions produced approximately the same R_t point estimates and 95% credible intervals (Figure S9).

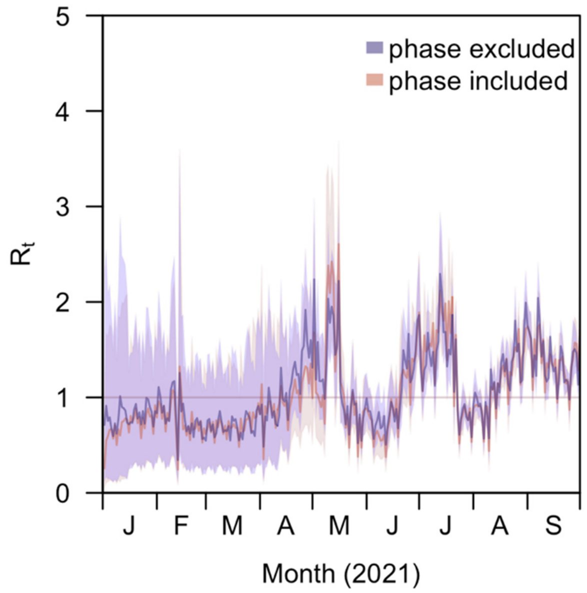

Figure S9. comparison of R_t estimates in scenarios when dormitory cases were or were not regarded as part of the imported case counts for Singapore.

Comparison of Models Utilizing Different Covariates in the *X* Matrix

Table S6. Deviance Information Criterion (DIC) of models including (i) all the available factors, (ii) mobility and epidemiological factors, (iii) 'retail and recreation' and 'residential' from google mobility data, vaccination rate, 13 of the indicator covariates in Oxford policy data with 'testing policy' and 'vaccination policy' excluded.

Region	NSW	NZ	SG	TW
**Full model**	1240	474	1586	1298
**Policy-excluded model**	1257	473	1623	1308
**Hybrid model**	1256	533	1591	1312

Table S7. Mean absolute difference between posterior median of R_t estimates given by the model including all the available factors and those given by the model including (i) mobility and epidemiological factors, (ii) 'retail and recreation' and 'residential' from google mobility data, vaccination rate, 13 of the indicator covariates in Oxford policy data with 'testing policy' and 'vaccination policy' excluded.

Region	NSW	NZ	SG	TW
**Full model v.s. Policy-excluded model**	0.23	1.00	0.13	0.42
**Full model v.s. Hybrid model**	0.20	0.29	0.47	0.66

Table S8. Smoothness (average absolute difference between neighbouring values) of posterior median of R_t estimates given by the model including (i) all the available factors, (ii) mobility and epidemiological factors, (iii) 'retail and recreation' and 'residential' from google mobility data, vaccination rate, 13 of the indicator covariates in Oxford policy data with 'testing policy' and 'vaccination policy' excluded.

Region	NSW	NZ	SG	TW
**Full model**	0.25	0.27	0.21	0.26
**Policy-excluded model**	0.15	0.28	0.19	0.30
**Hybrid model**	0.14	0.06	0.11	0.20

Comparison of Predictions for Scenarios Allowing Different Time Lags

Considering possible delays in report and assume the regression function as

$$R_t = \boldsymbol{X}_{t-d}\boldsymbol{\beta} + \alpha,$$

where $d \in \mathbb{N}$ is the candidate time lag, we did predictions of the next-day case counts by using an inference window of 90 days for the four different regions. We compared the true values with 50% and 95% credible interval of the posterior predictive distribution of the case counts and calculated the proportion of real values that fell in the two intervals respectively. Note that proportions of real case counts that fell within the credible intervals of posterior predictive distributions might be larger than they should be, which was due to the large number of virtually zero case counts that were always covered in both 50% and 95% credible intervals. We saw no significant differences in the four different scenarios for the four regions, but the optimal choice of time lags for each of them varied.

Table S9. Forecasting accuracy when allowing different times of delays in report for the four regions.

Time Lag, d	Predictions in 50% CrI (NSW)	Predictions in 95% CrI (NSW)	Predictions in 50% CrI (NZ)	Predictions in 95% CrI (NZ)	Predictions in 50% CrI (SG)	Predictions in 95% CrI (SG)	Predictions in 50% CrI (TW)	Predictions in 95% CrI (TW)
**0**	71%	96%	86%	98%	63%	95%	80%	98%
**1**	71%	94%	86%	97%	63%	95%	77%	97%
**2**	73%	93%	86%	96%	64%	95%	79%	95%
**3**	71%	96%	87%	97%	58%	93%	77%	95%
**4**	72%	96%	86%	97%	61%	92%	74%	95%
